# Rhamnogalacturonan‐I is a determinant of cell–cell adhesion in poplar wood

**DOI:** 10.1111/pbi.13271

**Published:** 2019-10-23

**Authors:** Haibing Yang, Matheus R. Benatti, Rucha A. Karve, Arizona Fox, Richard Meilan, Nicholas C. Carpita, Maureen C. McCann

**Affiliations:** ^1^ Department of Biological Sciences Purdue University West Lafayette IN USA; ^2^ Department of Forestry and Natural Resources Purdue University West Lafayette IN USA; ^3^ Purdue Center for Plant Biology West Lafayette IN USA; ^4^ Department of Botany and Plant Pathology Purdue University West Lafayette IN USA; ^5^ Present address: Arcadis U.S., Inc 150 West Market St., Suite 728 Indianapolis IN 46204 USA

**Keywords:** cell–cell adhesion, rhamnogalacturonan‐I, rhamnogalacturonan lyase, middle lamella, poplar, xylan, lignin, cell wall

## Abstract

The molecular basis of cell–cell adhesion in woody tissues is not known. Xylem cells in wood particles of hybrid poplar (*Populus tremula* × *P. alba* cv. INRA 717‐1B4) were separated by oxidation of lignin with acidic sodium chlorite when combined with extraction of xylan and rhamnogalacturonan‐I (RG‐I) using either dilute alkali or a combination of xylanase and RG‐lyase. Acidic chlorite followed by dilute alkali treatment enables cell–cell separation by removing material from the compound middle lamellae between the primary walls. Although lignin is known to contribute to adhesion between wood cells, we found that removing lignin is a necessary but not sufficient condition to effect complete cell–cell separation in poplar lines with various ratios of syringyl:guaiacyl lignin. Transgenic poplar lines expressing an *Arabidopsis thaliana* gene encoding an RG‐lyase (*AtRGIL6*) showed enhanced cell–cell separation, increased accessibility of cellulose and xylan to hydrolytic enzyme activities, and increased fragmentation of intact wood particles into small cell clusters and single cells under mechanical stress. Our results indicate a novel function for RG‐I, and also for xylan, as determinants of cell–cell adhesion in poplar wood cell walls. Genetic control of RG‐I content provides a new strategy to increase catalyst accessibility and saccharification yields from woody biomass for biofuels and industrial chemicals.

## Introduction

Biomass recalcitrance generally refers to the molecular interactions between lignin and carbohydrate to reduce the yields of sugar by cellulolytic enzymes (Himmel *et al.*, 2007). However, at the tissue scale, differing proportions of cell types, sizes and shapes, and sites of cell–cell adhesion, impart emergent biophysical properties affecting both normal plant growth and development, and the utilization of biomass for conversion to biofuels. Thus, we have broadly defined biomass recalcitrance as those features that disproportionately increase energy requirements, increase cost and complexity of biorefinery operations and/or reduce the conversion of biomass carbon into desired products (McCann and Carpita, 2015). Particle size is a critical factor for efficient heat transfer and catalyst accessibility for thermochemical and/or biochemical conversion of lignocellulosic biomass to fuels or chemicals (Viamajala *et al.*, 2009). Comminution, the mechanical reduction of intact biomass to particles, is an energy‐intensive process – the smaller the particle, the greater the energy consumption (Miao *et al.*, 2011). By determining the molecular basis for cell–cell adhesion in poplar wood, we sought to identify potential targets for genetic modifications that enhanced cell–cell separation during comminution. Genetic modification that results in reduction in particle size of bioenergy crops at the point of use could substantially reduce the energy inputs for biofuel and chemical production.

The biomechanical properties of tissues and organs depend on the strength and nature of molecular interactions at the interfaces between cells (Zamil and Geitmann, 2017). In parenchyma of fruits and some vegetables, cells adhere to each other at a pectin‐rich interface called the middle lamella. Disruption of calcium and ester cross‐links with chemical reagents causes cell–cell separation in these tissues by breaking the molecular bonds between pectins (Marry *et al.*, 2006; Ordaz‐Ortiz *et al.*, 2009). Pectins comprise a class of acidic polysaccharides including homogalacturonans (HGs), polymers of galacturonic acid (GalA) and rhamnogalacturonan (RG)‐I, a pectic polymer of alternating rhamnose (Rha) and GalA residues substituted with side chains of arabinans and galactans. Calcium cross‐linked HGs are enriched in middle lamellae. RG‐II is a distinct domain of HG, substituted with complex Rha‐containing chains, and can dimerize through borate di‐di‐ester cross‐links (O’Neill *et al.*, 2004). As HG might be covalently linked to RG‐I and RG‐II, these molecules might also contribute to cell–cell adhesion (Tan *et al.*, 2013; Vincken *et al.*, 2002). However, the molecular determinants of cell–cell adhesion in woody tissues and organs are not known. The interfaces between xylem cells of wood are elaborated into a compound middle lamella (CML) containing pectins, cellulose and hemicelluloses, in addition to lignin and other phenolic substances (Donaldson, 2001; Fromm *et al.*, 2003; Westermark, 1985).

In poplar (*Populus* spp.) and Arabidopsis (*Arabidopsis thaliana*), manipulation of lignin composition by altering expression of genes in the phenylpropanoid pathway has provided materials with various ratios of syringyl (S) to guaiacyl G, monolignols (Coleman *et al.*, 2008; Huntley *et al.*, 2003; Min *et al.*, 2013; Smith *et al.*, 2015; Wang *et al.*, 2018; Weng and Chapple, 2010). Characterization of cell structure in woody material by electron microscopy showed that cell separation occurred in genetic variants with high S‐lignin after removal of xylan, a (1→4)‐β‐linked polymer of xylose, using maleic acid catalysis (Ciesielski *et al.*, 2014). These results suggest that both lignin and xylan independently, and perhaps their interactions, have important roles in cell–cell adhesion. However, pectins might also be extracted by maleic acid (Kim *et al.*, 2012; Smith *et al.*, 2015).

In this study, we used chemical and enzymatic treatments sequentially to remove lignin, xylan and/or pectin from the CML of wild type (WT, *Populus tremula *× *P. alba* cv. INRA 717‐1B4) and genetic variants of hybrid poplar, and measured the release of cells from finely milled‐wood particles. Using transgenic lines with various S:G ratios, we observed that de‐lignification was not sufficient to disrupt cell–cell adhesion, regardless of lignin composition. However, high‐S‐lignin genotypes fragmented to single cells and small cell clusters more easily than WT or high‐G‐lignin genotypes. Xylan comprised over 90% of the carbohydrate extracted during cell–cell separation, but sugar and methylation analyses indicated that RG‐I, was also removed. Treatment of de‐lignified wood particles with both xylanase and RG‐lyase enzymatic activities was required to achieve complete cell–cell separation. RG‐lyases cleave the backbone of RG‐I (Mutter *et al.*, 1998; Oomen *et al.*, 2002). Hydrolysis or lysis of HG did not facilitate cell–cell separation. To test the role of RG‐I in cell–cell adhesion, we generated transgenic poplar lines that expressed the *A. thaliana RG‐lyase6* (*AtRGIL6*) gene. These transgenic lines showed increased cell–cell separation in chemical, enzymatic and mechanical assays. Genetic control of RG‐I content provides a new strategy to increase catalyst accessibility and saccharification yields at the molecular level, and to increase fragmentation of woody biomass at the tissue and organ level.

## Results

### De‐lignification of poplar particles is necessary but not sufficient to disrupt cell–cell adhesion

**Figure 1:**
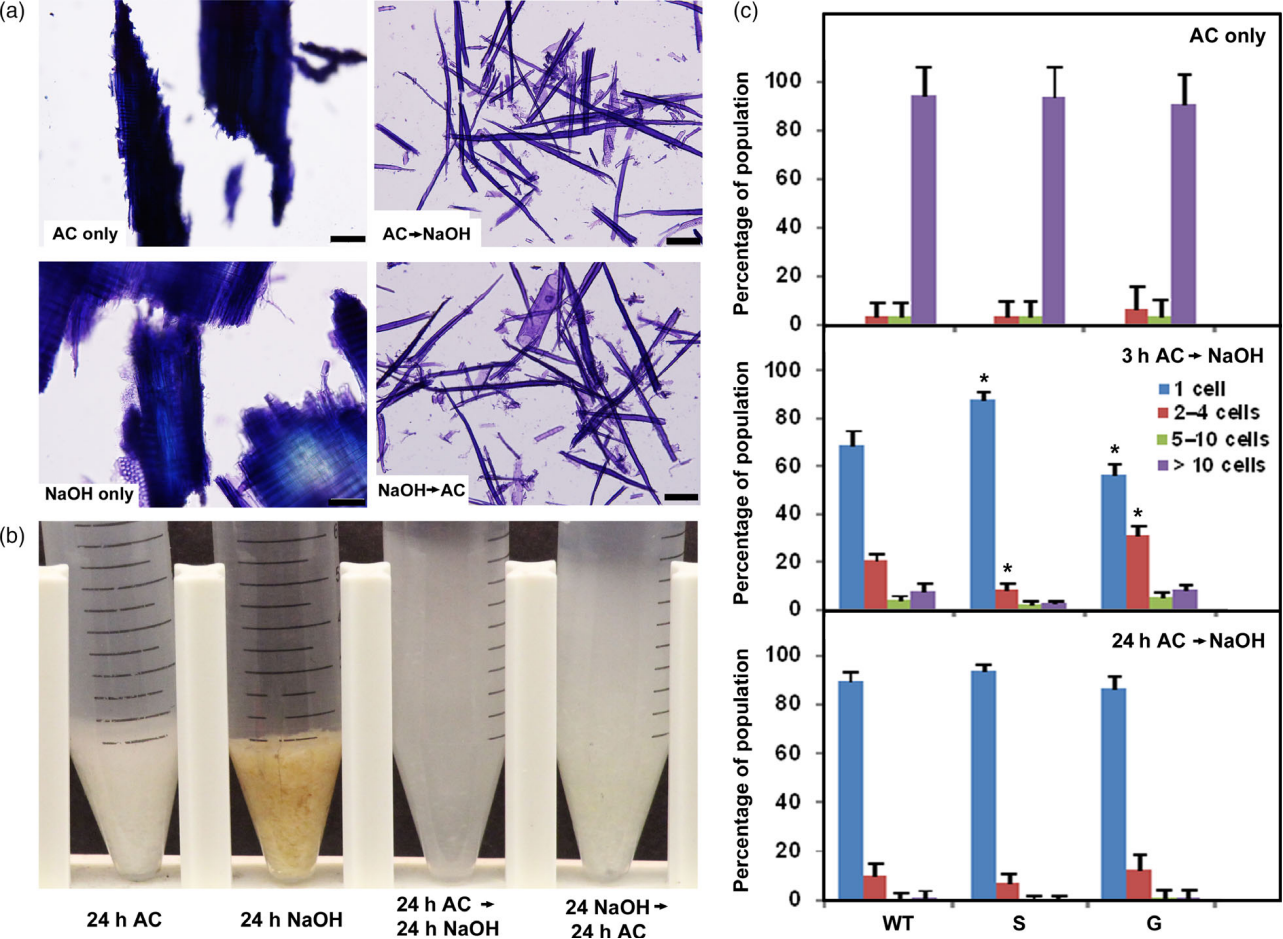
Cell–cell separation of poplar wood particles after sequential extraction with acidic chlorite and dilute alkali. (a) Bright‐field micrographs of toluidine blue‐stained wild‐type (WT) 717‐1B4 particles with acidic chlorite alone for 24 h (AC only); 0.1 m NaOH, dilute alkali, treatment alone for 24 h (NaOH only); 24 h acidic chlorite followed by 24 h dilute alkali (AC→NaOH); and 24 h dilute alkali followed by 24 h acidic chlorite (NaOH→AC). Bar, 100 µm. (b) Micrographs of settled‐cell volumes of wood particles in 15‐mL Falcon tubes after treatments as indicated. (c) Percentages of cells released from WT and lignin genetic variants as single cells or cell clusters. Top: treatment of WT, high‐S lignin (S) and high‐G‐lignin G, variants with acidic chlorite alone for 24 h (AC only). Middle: treatment with acidic chlorite for 3 h, followed by 0.1 m NaOH for 24 h (3h AC→NaOH). Bottom: treatment with acidic chlorite for 24 h, followed by 0.1 m NaOH. Percentages of single cells (blue) or cell clusters of 2‐4 cells (red), 5‐10 cells (green), and> 10 cells (purple) were determined from >1000 cells counted per genotype/treatment. Values are the means ± SD (*n* = 3 technical replicates); asterisks indicate significant differences based on Tukey‐Kramer Post Hoc test after one‐way ANOVA, *P* ≤ 0.05 relative to WT.

To determine the polymers that contribute to cell–cell adhesion in hybrid poplar wood, we explored several chemical and enzymatic treatments that differentially extract or cleave cell‐wall constituents. Three‐year‐old poplar stems were debarked, lyophilized and milled; the resulting particles were sieved to between 300 µm and 1 mm in size. As a crude measure of the extent of cell–cell separation, we used an assay developed by Ordaz‐Ortiz *et al. *(2009), who showed that settled‐cell volumes were correlated with the extent of cell–cell separation. We also counted the number of single cells and cells in small clumps, stained with toluidine blue, using bright‐field microscopy. Chemical reagents known to chelate calcium or de‐esterify pectins, or treatment with enzymes that cleave HG or xylan, had no effect on milled WT poplar material. Exposure to warm 88 mm acidified sodium chlorite for 24 h, a treatment that oxidizes lignin and other phenylpropanoid substrates (Carpita, 1983; Collings *et al.*, 1978; Reeves, 1987), did not result in cell separation from the particles (Figure 1a) or an increase in settled‐cell volume (Figure 1b). Particles treated with 0.1 m NaOH at ambient temperature for 24 h, a treatment that extracts pectin and weakly bound hemicellulosic substances (Carpita, 1983; Macdonald *et al.*, 1983; Sun *et al.*, 2001), resulted in some fraying of particle ends but little cell separation or change in settled volumes (Figures 1a,b). However, sequential treatments with warm acidic chlorite for 24 h and dilute alkali for 24 h, regardless of order, resulted in almost complete cell separation and a large increase in settled‐cell volumes (Figure 1).

**Figure 2:**
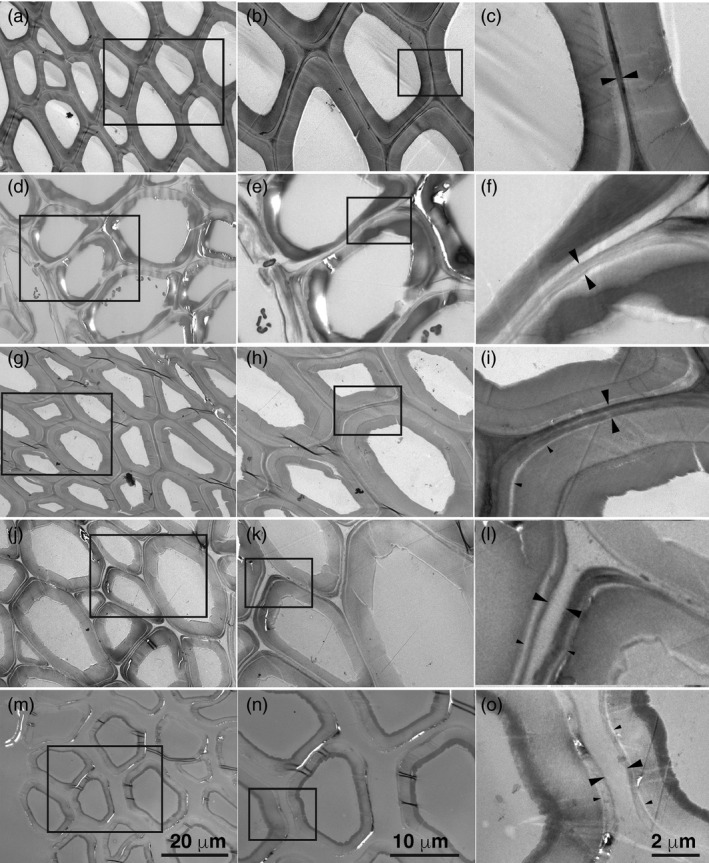
Electron micrographs of untreated and treated WT poplar particles. (a) Electron micrographs of untreated WT particles. Bar, 20 µm. (b) Magnification of inset box in (a). Bar, 10 µm. (c) Magnification of inset box in (b). Large arrowheads indicate middle lamella. Bar, 2 µm. (d) WT particles after treatment with acidic chlorite (AC) for 24 h. Bar, 20 µm. (e) Magnification of inset box in (d). Bar, 10 µm. (f) Magnification of inset box in (e). Large arrowheads indicate middle lamella. Small arrowheads indicate a zone of reduced electron density between the primary and secondary wall. Bar, 2 µm. (g) WT particles after treatment with 0.1 m NaOH for 24 h. Bar, 20 µm. (h) Magnification of inset box in G. Bar, 10 µm. (i) Magnification of inset box in (h). Large arrowheads indicate middle lamella. Small arrowheads indicate a zone of reduced electron density between the primary and secondary wall. Bar, 2 µm. (j) WT particles after treatment with 3 h acidic chlorite followed by 24 h dilute alkali. Bar, 20 µm. (k) Magnification of inset box in (j). Bar, 10 µm. (l) Magnification of inset box in (k). Large arrowheads indicate middle lamella. Small arrowheads indicate a zone of reduced electron density between the primary and secondary wall. Bar, 2 µm. (m) WT particles after treatment with 24 h acidic chlorite followed by 24 h dilute alkali. Bar, 20 µm. (n) Magnification of inset box in (m). Bar, 10 µm. (o) Magnification of inset box in (n). Large arrowheads indicate middle lamella. Small arrowheads indicate a zone of reduced electron density between the primary and secondary wall. Bar, 2 µm.

Electron‐dense material was present in the middle lamellae and cell corners of untreated particles (Figure 2a‐c) and persisted in particles treated with acidic chlorite (Figure 2d‐f) or with dilute alkali (Figure 2g‐i). Secondary walls and cell corners of acidic chlorite‐treated particles showed patches of reduced staining, consistent with removal of lignin (Figure 2d‐f). The interface between the primary wall and the secondary wall also showed some evidence of separation in dilute alkali‐treated particles (Figure 2g‐i). Only upon sequential treatment of particles with acidic chlorite for 3 h (Figures 2j‐l), or 24 h (Figures 2m‐o), followed by dilute alkali, was material in the middle lamellae removed. The primary wall remained surrounding the secondary wall (Figure 2j‐o).

### High‐S‐lignin composition enhances the extent of cell–cell separation

We then tested the influence of lignin composition on cell–cell separation of particles treated with acidic chlorite and dilute alkali. A range of S:G ratios has been generated in transgenic poplar lines in which: (1) an *A. thaliana Ferulate‐5‐hydroxylase* (*AtF5H1*) was over‐expressed to increase the proportion of S‐lignin; and (2) poplar *F5H* expression was down‐regulated using RNA interference (RNAi) to increase the proportion of G‐lignin (Yang *et al.*, 2019) (Table S1). The WT and lignin genetic variants contained ~55–60% cellulose and ~33% lignin by weight (Figure S1). Treatment with acidic chlorite for 3 h resulted in loss of ~30% of Klason lignin content from all genotypes; 24‐h treatment removed ~60% of lignin content from WT and high‐S genotypes, and ~50% from high‐G‐lignin line (Figure S1). Subsequent treatment with 0.1 m NaOH resulted in removal of ~20% of the extracted dry weight as pectic and hemicellulosic material (Figure S1).

Acidic chlorite treatments limited to 3 h resulted in variation in size of cell clusters; the extent of cell separation was quantified and results were assigned to one of the following categories: single cells, and clusters of 2‐4, 5‐10, and> 10 cells. Limited chlorite treatments before alkali yielded 90% single cells with the remainder in clusters of 2–4 cells from the high‐S‐lignin genotype, whereas WT released 70% single cells, with the remainder in larger clusters (Figure 1c; Figure S2). The high‐G‐lignin genotype was more resistant to chlorite treatments, with single cells representing only 55% of the population (Figure 1c). Treatment with dilute alkali followed by acidic chlorite yielded similar results but with slightly lower proportions of single cells (Figures S2 and S3b).

Cell separation was nearly complete for all genotypes when acidic chlorite treatments were prolonged for 24 h before or after alkali treatment (Figures S2 and S3c). No single cells were observed in any genotype when particles were treated with acidic chlorite or dilute alkali alone, and ~95% of the cells remained in particles comprised of >10 cells (Figure 1c and Figure S3a).

### Xylan and RG‐I are extracted by acidic chlorite and dilute alkali

**Figure 3:**
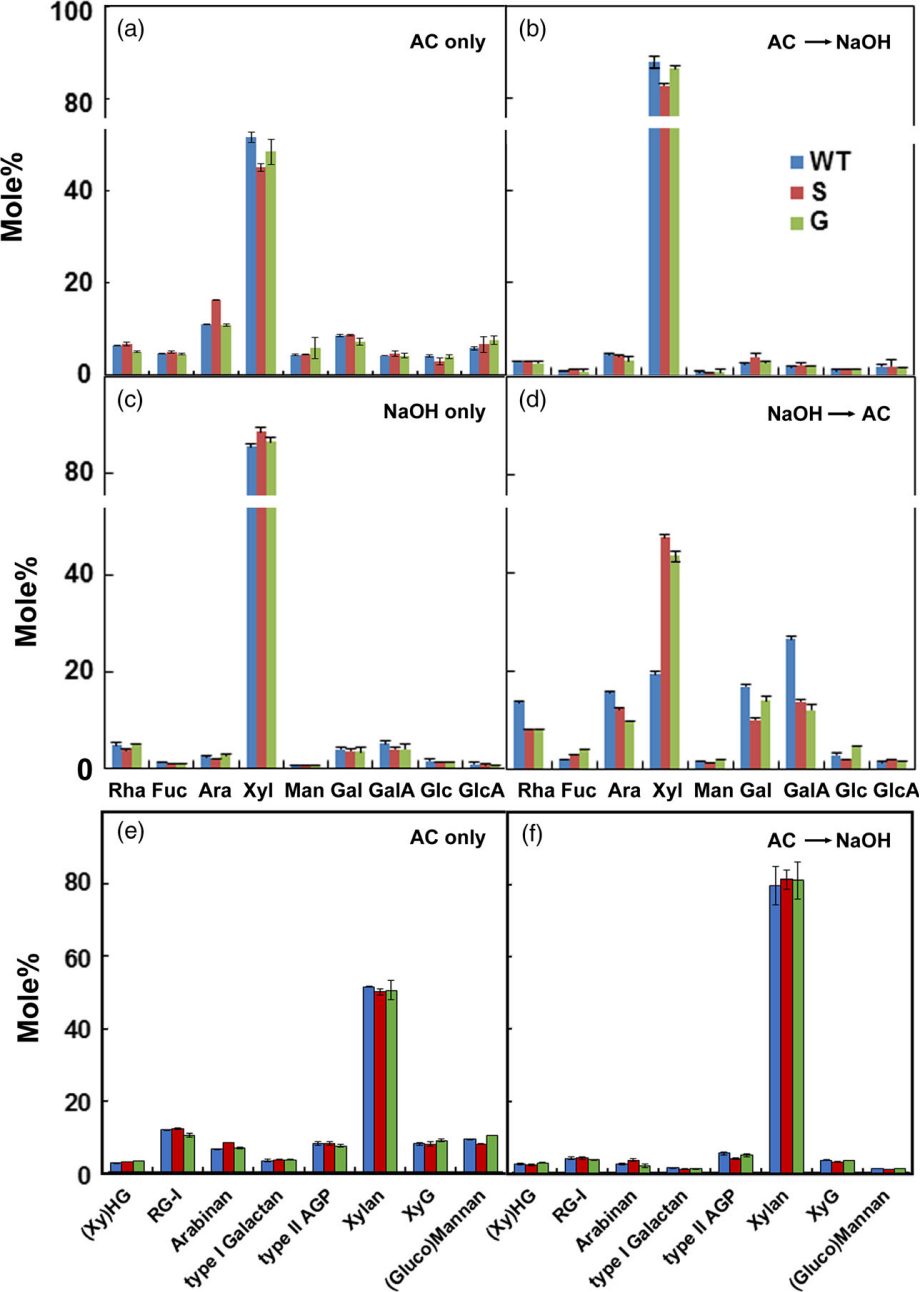
Monosaccharide and polysaccharide contents of materials extracted from wood particles of WT and lignin variants by acidic chlorite and dilute alkali. Monosaccharide distribution in materials extracted (a) by acidic chlorite alone for 3 h (AC only), (b) by 0.1 m NaOH for 24 h, following treatment with acidic chlorite for 3 h (AC→NaOH), (c) by 0.1 m NaOH alone for 24 h (NaOH only), and (d) by acidic chlorite for 3 h, following treatment with 0.1 m NaOH for 24 h (NaOH→AC). Relative proportions of major polysaccharides in extracts from (e) acidic chlorite (AC) treatment and (f) 0.1 m NaOH treatment after acidic chlorite treatment. Some polysaccharide names abbreviated as: xylogalacturonan, (Xy)HG; arabinogalactan protein, AGP; rhamnogalacturonan‐I, RG‐I; and xyloglucan, XyG. Values are derived from linkage analyses of cell walls isolated from wild type (blue), high‐S (red), and high‐G (green) lines.

Although the requirement for acidic chlorite demonstrated a role for lignin or phenylpropanoids in cell–cell adhesion in poplar, treatment with dilute alkali was also needed to effect complete cell separation. Chlorite treatment removed 2 to 2.4% of the initial dry weight as non‐cellulosic sugars, compared to 4 to 4.4% subsequently extracted by 0.1 m NaOH (Table S2), the concentration of alkali sufficient to cause cell separation. Little material was extracted from WT walls with less than 0.1 m NaOH, and most of the total carbohydrate remained in the pellet after treatment with 4 m NaOH; treatment with acidic chlorite rendered substantially more material extractable by 0.1 m or less NaOH (Figure S4). Acidic chlorite and 0.1 m NaOH treatments extracted Xyl with smaller amounts of Rha, Ara, Gal and GalA (Figure 3). The ratio of Rha:GalA in material extracted by acidic chlorite, and also in subsequent dilute alkali treatment, was 1:1 (Figure 3), indicating that mainly RG‐I is extracted. Methylation analysis of alkali‐extracted materials showed abundant 4‐Xyl and 2,4‐Xyl branch‐point residues, with associated *t*‐GlcA, confirming that glucuronoxylans constitute the major hemicellulosic polysaccharide (Figure 3e,f; Table S3). The 2‐ and 2,4‐Rha residues and ample 4‐GalA linkages indicated that both HG and RG‐I were also present in alkali extracts, together with 5‐linked arabinan and 4‐linked galactan side chains of RG‐I (Figures 3e,f). Although substantial amounts of *t*‐Xyl were found in the chlorite extractions, the low amounts of 4‐ and 4,6‐Glc residues indicate that little xyloglucan was present, and the *t*‐Xyl might, therefore, reflect non‐reducing terminal residues of short xylan chains, but more likely of 3,4‐GalA residues in xylogalacturonan. Amounts of 4‐Mannose (Man) and 4,6‐Man were negligible, indicating low content of gluco(galacto)mannans. Using diagnostic linkages to determine the relative proportions of the major polysaccharides in these extracts, we found that, regardless of order of extraction, acidic chlorite yields significant proportions of RG‐I and its side chains, and xylan, while alkali extracts primarily xylan, with RG‐I and a small amount of HG (Figure 3; Table S3).

### Xylan and RG‐I contribute to cell–cell adhesion in lignified wood cells

As glucuronoxylan was the major polysaccharide found in either the chlorite‐ or alkali‐soluble fraction, xylanase digestion followed by acidic chlorite was used to assay cell separation in WT and high‐S genotypes. Particles smaller than 300 µm were used to increase the surface area for enzyme accessibility. Consistent with the behaviour of large particles (Figure 1a,c), cell separation was not observed in these smaller particles with acidic chlorite treatment alone (Figure 3a). However, single cells of both genotypes represented ~ 80% of the population in acidic chlorite treatments when preceded by incubation with *Trichoderma longibrachiatum* endo‐(1→4)‐β‐d‐xylanase M3 (Figure S5).

As treatment with xylanase and acidic chlorite gave incomplete cell separation, we hypothesized that RG‐I and its side chains might also contribute to cell–cell adhesion. Treatment of milled poplar samples with an *Aspergillus niger* endo‐(1→5)‐α‐L‐arabinanase (arabinanase), an *A. aculeatus* endo‐(1→4)‐α‐D‐polygalacturonase (PGase), a *Clostridium thermocellum* endo‐(1→4)‐α‐D‐polygalacturonan pectate lyase (pectate lyase) or endo‐rhamnogalacturonan‐I lyase (RG‐lyase), followed by acidic chlorite alone, or by dilute alkali alone, resulted in little or no cell separation (Figure S6). Cell separation observed upon treatment with a combination of chlorite and alkali after digestion with arabinanase, PGase, a combination of pectin methyl esterase (PME) and PGase, or pectate lyase were indistinguishable from controls without enzyme. However, RG‐lyase treatment, prior to acidic chlorite for 3 h and dilute alkali for 24 h, resulted in separation to ~90% single cells, with the remainder in clusters of only 2 to 4 cells (Figures S6 and S7a). The amount of GalA released from pectins was not increased if particles were treated with PME and PGase, compared to PGase or pectate lyase alone (Figure S7b), and the degree of methyl esterification of cell walls was measured as 10%.

**Figure 4:**
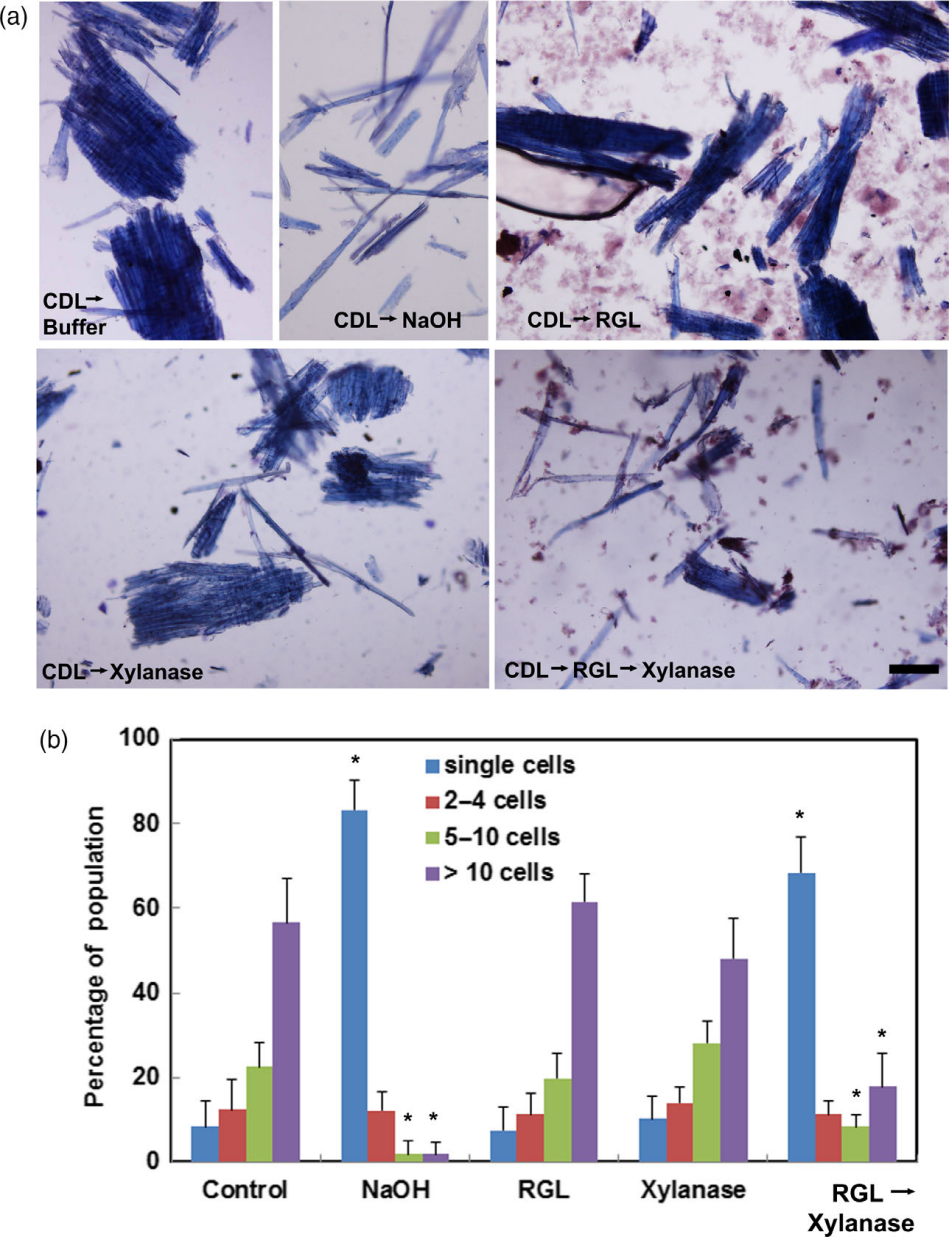
Cell–cell separation of Catalytically De‐Lignified (CDL) WT Poplar wood particles after dilute alkali or enzyme treatments. (a) Bright‐field micrographs of Toluidine Blue‐stained WT particles following treatment with Ni/C catalyst to remove lignin and then incubation in buffer (CDL→buffer); CDL followed by dilute alkali treatment for 24 h (CDL→NaOH); CDL followed by treatment with endo‐(1→4)‐α‐rhamnogalacturonan‐I lyase (CDL→RGL); CDL followed by treatment with endo‐(1→4)‐β‐D‐xylanase (CDL→xylanase): CDL followed by treatment with endo‐(1→4)‐α‐rhamnogalacturonan‐I lyase and then endo‐(1→4)‐β‐D‐xylanase (CDL→RGL→xylanase). Bar, 100 µm. (b) Percentages of single cells (blue) or cell clusters of 2‐4 cells (red), 5–10 cells (green) and> 10 cells (purple) were determined from >1000 cells counted per treatment. Values are the means ± SD (*n* = 3 technical replicates); asterisks indicate significant differences based on Tukey‐Kramer Post Hoc test after one‐way ANOVA, *P* ≤ 0.05.

As an alternative to acidic chlorite, a metallic Ni/C catalyst was used to de‐lignify poplar wood particles (Luo *et al.*, 2016). De‐lignification followed by either RG‐lyase or xylanase treatment alone resulted in little cell separation, whereas the combination of RG‐lyase and xylanase treatments induced cell separation in the de‐lignified wood almost as effectively as alkali treatment (Figure 4).

### Reducing RG‐I content by expressing an RG‐lyase in poplar facilitates cell separation

**Figure 5:**
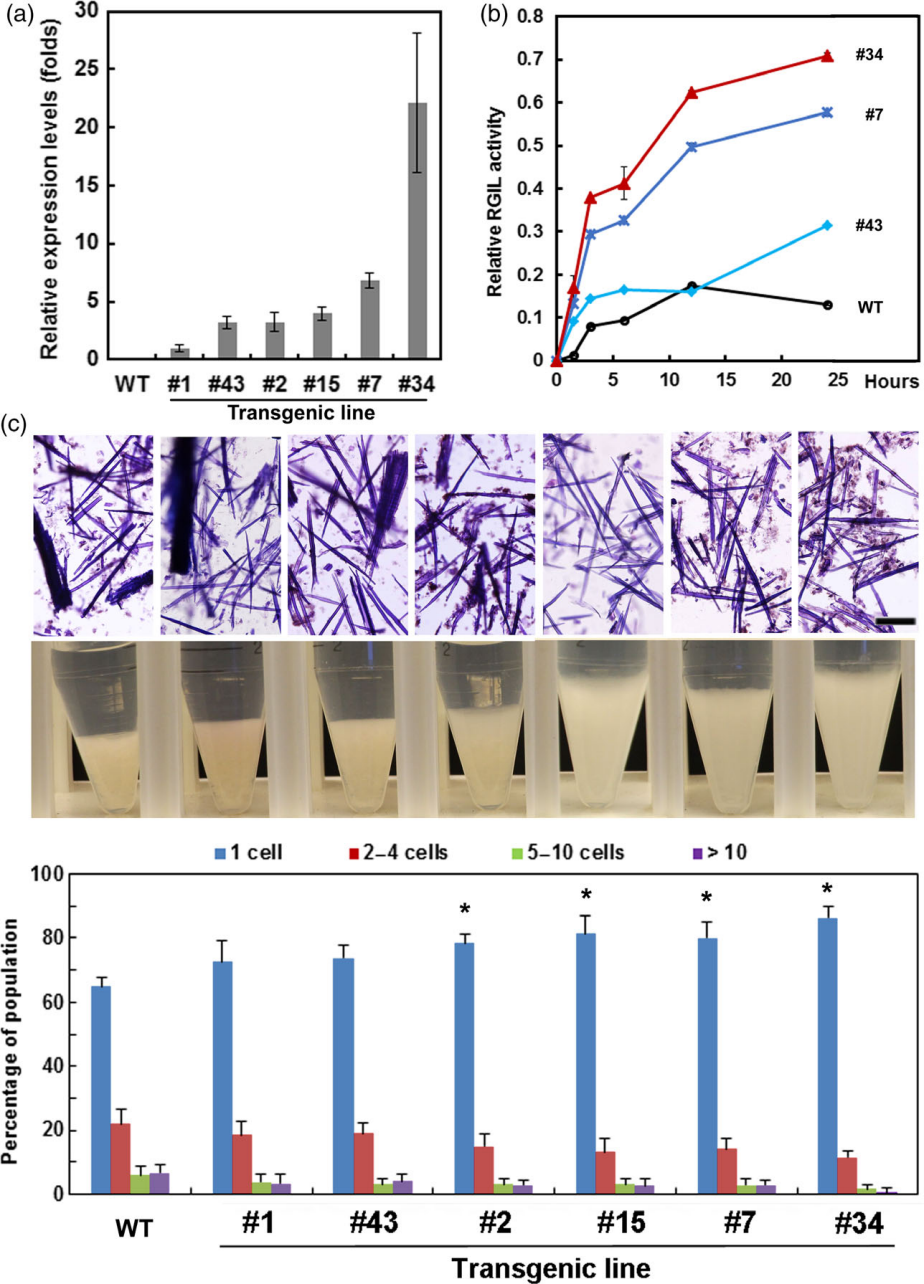
Cell–cell separation of WT and *AtRGIL6*‐expressing poplar wood particles after sequential extraction using acidic chlorite and dilute alkali. (a) Relative expression levels of the transgene in six independent *AtRGIL6*‐expressing poplar lines as determined by qRT‐PCR, normalized to line #1. Values are the means ± SD, *n* = 3 biological replicates. (b) Time‐course of relative RG‐lyase activity of cell‐wall proteins extracted from wild type (WT) and three *AtRGIL6*‐expressing lines. Values are the means ± SD, *n* = 3 biological replicates. (c) Top: bright‐field micrographs of Toluidine Blue‐stained WT and particles from six independent transgenic lines following treatment with acidic chlorite for 3 h followed by 0.1 m NaOH for 24 h. Bar, 200 µm. Middle: Photographs of settled‐cell volumes of wood particles in 15‐mL Corning tubes. Bottom: Percentages of single cells (blue) or cell clusters of 2‐4 cells (red), 5‐10 cells (green), and >10 cells (purple) were determined from> 1000 cells counted per genotype/treatment. Values are the means ± SD (*n* = 3 biological replicates); asterisks indicate significant differences based on Student t‐test *P* ≤ 0.05 relative to WT.

**Figure 6:**
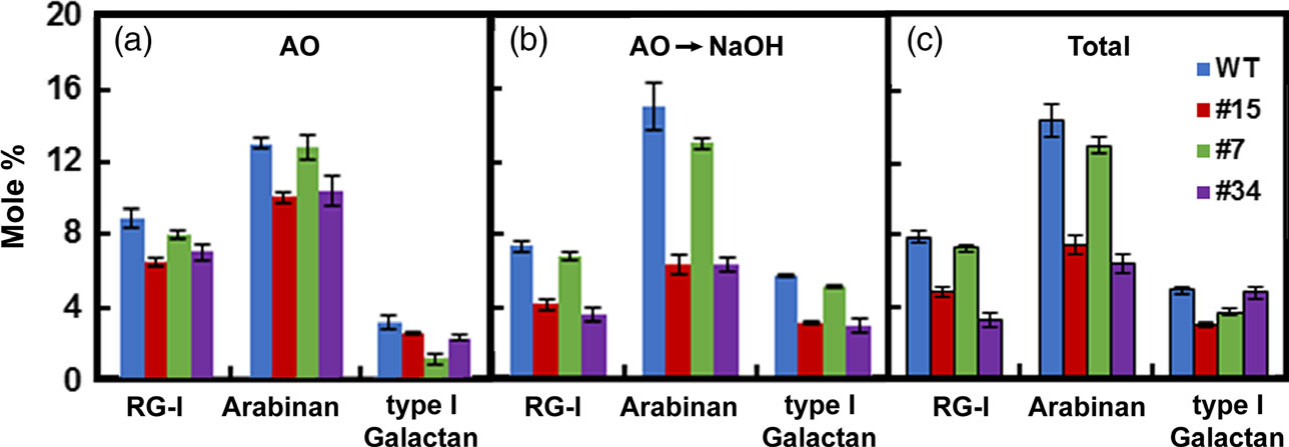
Mole percentages of cell‐wall RG‐I and side chains in materials extracted from poplar wood particles of WT and three independent *AtRGIL6*‐expressing lines. Relative proportions of RG‐I and arabinan and galactan side chains extracted by (a) ammonium oxalate (AO), (b) dilute alkali, after ammonium oxalate treatment (AO→NaOH), and (c) ammonium oxalate‐ and NaOH‐extracted material (Total). Values are derived from cell walls isolated from wild type (blue) and *AtRGIL*‐expressing lines #15 (red), #7 (green) and #34 (purple), and represent means ± SD (*n* = 3 biological replicates).

Because RG‐lyase promoted cell separation after treatments that removed lignin and xylan, we expressed the *AtRGIL6* gene under the control of a constitutive promoter in WT poplar. Over 30 lines were regenerated; we selected six that exhibited a range of transgene expression levels (1‐ to 20‐fold, relative to lowest expressing line #1) (Figure 5a). Variations in stem length, stem diameter and number of leaves were not correlated with transcript abundance of the transgene (Figure S8). RG‐lyase activity was detectable in WT indicating expression of one or both *PtRGIL* endogenous sequences. However, total RG‐I lyase activity was greater in the isolated cell‐wall‐protein fraction from high‐expressing lines #7 and #34, whereas low‐expressing line #43 showed similar activity to WT (Figure 5b). Using cell‐wall proteins isolated from line #34, the extract had highest activity at pH 5 and displayed higher activity towards RG‐I from *A. thaliana* seed mucilage than other RG‐I substrates (Figure S9). We isolated cell walls from WT and lines #15, #7 and #34, and extracted them with ammonium oxalate and dilute alkali to enrich the pectin moiety in fractions for sugar and linkage analyses (Figure S10). From the mole % values of diagnostic linkages, the total content of RG‐I in these fractions was reduced from 8 % in WT to an average of 6 % in the high‐*RGIL6*‐expressing lines (Figure 6). The ratio of 2,4‐Rha to 2‐Rha indicated that RG‐I branching was slightly increased in the transgenic lines (average ~ 1:2) compared to WT (~ 1:1.7) (Table S4). The extent of cell separation in *AtRGIL6*‐expressing lines was correlated with an increase in settled‐cell volume and showed enhanced cell–cell separation, as quantified by released cells and cell clusters (Figure 5c).

**Figure 7:**
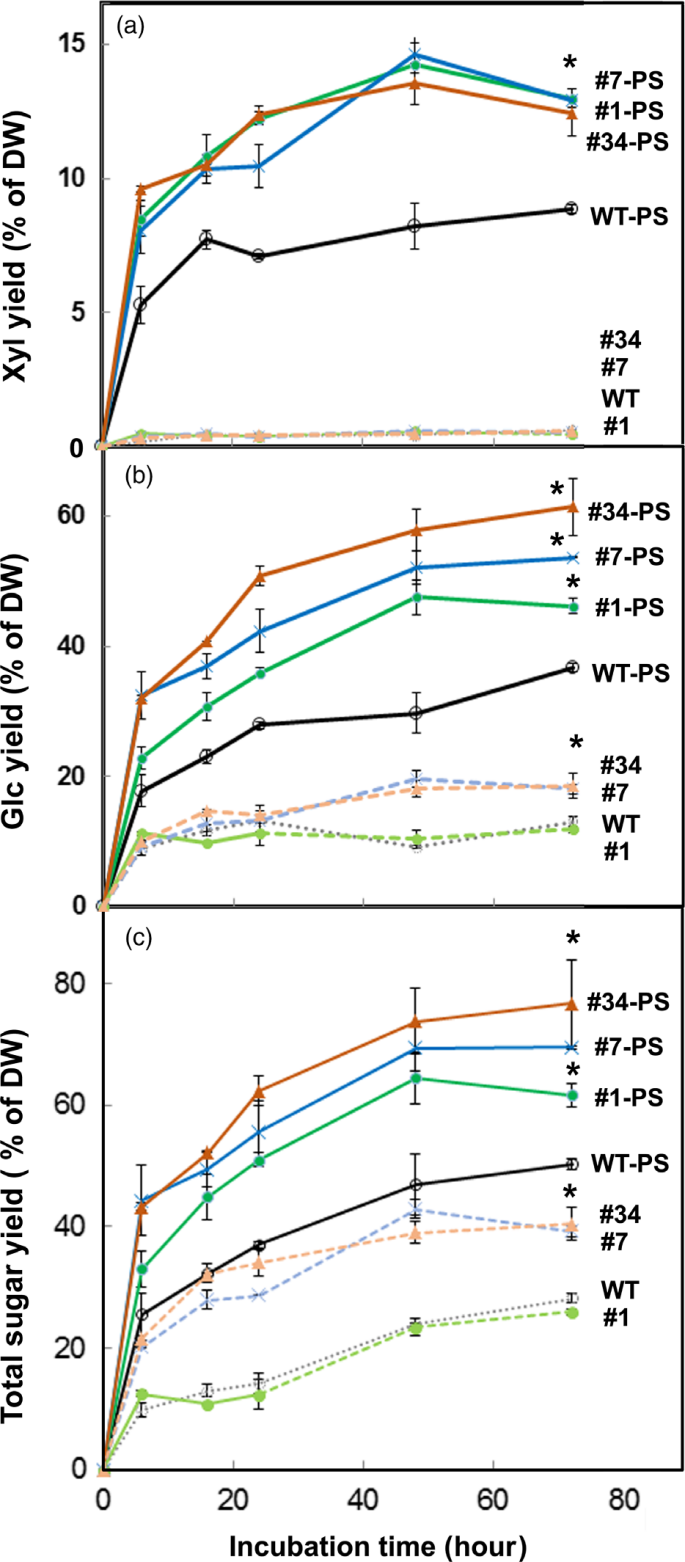
Expression of *AtRGIL6* in WT poplar facilitates enzymatic digestion. Wood particles from wild type (WT) and three *AtRGIL6*‐expressing lines (#1, #7, and #34) were treated with acidic chlorite for 3 h followed by 0.1 m NaOH for 24 h to generate samples with partial cell separation (WT‐PS, #1‐PS, #7‐PS, #34‐PS) before incubation with a Cellic^TM^ Ctec2 enzyme cocktail. Dotted lines represent sugar yields with a Cellic^TM^ Ctec2 enzyme cocktail in untreated controls. Yields are shown as a percentage of initial dry weight (DW). Values are the means ± SD, *n* = 3 biological replicates. Time‐course of yields in 72 h of (a) xylose, (b) glucose, and (c) total sugar.

We tested cell‐wall cellulose and xylan digestibility, with the Cellic^TM^ Ctec2 enzyme cocktail of glucanase and xylanase activities, of untreated lines and from partially de‐lignified particles (3 h acidic chlorite) treated with dilute alkali. Xylose yield from all genotypes was low (Figure 7a). Expression of *AtRGIL6* increased glucose yield in high‐expressing lines #7 and #34 by ~25% relative to untreated WT and low‐expressing line #1 (Figure 7b). After partial cell separation treatments, glucose yield increased from ~35% in WT and line #1 to ~50% dry weight in *AtRGIL6*‐expressing lines #7 and #34 (Figure 7b). Xylose yields also increased from ~8% in WT to ~12% in the transgenic lines (Figure 7a), contributing to total sugar yields (Figure 7c).

We compared the fragment sizes resulting from mechanically grinding dry wood particles of a defined size range for 30 s using a cell disruptor in which stainless steel balls are agitated to disrupt tissues (Figure S11). High‐expressing lines #7 and #34 yielded smaller particles than WT, with line #15 generally intermediate for equivalent energy inputs (Figure S11), indicating that biomechanical properties of the particles were correlated with the level of transgene expression.

## Discussion

Our poplar transgenic lines, in which the activity of RG‐lyase and, therefore, the amount of its substrate is modulated, provide new tools for dissecting the function of RG‐I in cell‐wall architecture and in plant growth. Various side chains of RG‐I have been implicated in the mechanical properties of pea (*Pisum sativum*) cotyledon cell walls (McCartney *et al*, 2000); the softening of apples (*Pyrus malus*) during fruit ripening (Peña and Carpita, 2004); loosely attached constituent cells in tobacco (*Nicotiana plumbaginifolia*) calli (Iwai *et al.*, 2001); wall porosity (Orfila and Knox, 2000; Oxenbøll‐Sørensen *et al.*, 2000); and potato (*Solanum tuberosum*) cell elongation and proliferation (Bush *et al.*, 2001). Transgenic potato tubers, in which fungal arabinanase or galactanase enzymes have been over‐expressed to degrade arabinan or galactan side chains, respectively, are as strong as control tubers but lose elasticity, becoming brittle (Ulvskov *et al.*, 2005). Digestion of the RG‐I backbone by expression of a fungal RG‐lyase resulted in cell swelling in the cortex and periderm of the tuber (Oomen *et al.*, 2002). In strawberry (*Fragaria x ananassa*), knockdown of RG‐lyase expression is correlated with reduced dissolution of the middle lamella during fruit ripening (Molina‐Hidalgo *et al.*, 2013). Over‐expression of a *Solanum lycopersicon* homolog resulted in increased tomato fruit firmness (Ochoa‐Jiménez *et al.*, 2018). In solution, neutral sugar side chains bind water and increase solubility (Belton, 1997) and reduce intrinsic viscosity, but contribute to polymer entanglement, resulting in higher viscosity at higher RG‐I concentrations (Hwang *et al.*, 1993). In principle, different micro‐environments might be created within cell walls by altering the structure of RG‐I and its side chains to regulate wall properties, such as porosity, water‐binding or resistance to compressive forces.

In poplar wood, we estimated the weight proportion of RG‐I to be ~2%, based on the proportion of non‐cellulosic sugars by mass balance (Table S2), and the relative mole% of RG‐I and its side chains represented by non‐cellulosic sugars (Figure S4). We observed an increased propensity of the tissue to fragment when the abundance of RG‐I is further reduced in transgenic lines. RG‐I isolated from flax (*Linum usitatissimum*) gelatinous fibres forms hydrogels with hyperelastic properties (Mikshina *et al.*, 2015). We hypothesize that RG‐I imparts elasticity to the CML, important for expansion and contraction of woody tissues in response to temperature shifts.

### Modifying the S:G ratio of lignin impacts cell–cell adhesion

In woody species, lignification is initiated in, and propagates from, the CML (Donaldson, 2001; Zamil and Geitmann, 2017). Consequently, treatments such as acidic chlorite would be expected to disrupt cell–cell adhesion. Acidic chlorite treatments are also known to increase sugar release, upon digestion with hydrolytic enzymes, from Zinnia tracheary elements differentiated *in vitro* (Lacayo *et al.*, 2013), on sugarcane bagasse (Siqueira *et al.*, 2013), and on a variety of other woody grasses (Reeves, 1987), perhaps because of increased accessibility to glucanases as a result of de‐lignification. Cell separation would also be expected as a result of de‐lignification using the Ni/C chemical catalyst (Luo *et al.*, 2016). Although material was extracted from some cell corners of acidic chlorite‐treated particles (Figures 2d‐f), the middle lamellae remained and were only removed after sequential extraction with dilute alkali (Figure 2j‐o). Although genetic modification of lignin has caused severe growth and developmental phenotypes (Jones *et al.*, 2001; Schilmiller *et al.*, 2009), high‐S lignin variants exhibit little or no disruption of normal growth in *A. thaliana* (Franke *et al.*, 2000; Marita *et al.*, 1999; Meyer *et al.*, 1998), or in the poplar variants described here. High‐S poplar, with similar total lignin content, displayed increased cell–cell separation relative to WT and high‐G‐lignin variants, with 3‐h acidic chlorite and 24‐h dilute alkali treatments (Figure 1). The highly methylated structure of S‐lignin might contribute less cross‐linking within the CML, or changes in lignin structure might reduce cross‐linking of other cell‐wall components. With 24‐h treatments of acidic chlorite and dilute alkali, cell separation was complete for all genotypes. The requirement for subsequent treatment with dilute alkali shows that de‐lignification is a necessary but not sufficient condition to separate woody particles into single cells and that cell–cell separation occurs with the loss of material from middle lamellae.

### Xylan and RG‐I contribute to cell–cell adhesion between poplar wood cells

Electron‐dense stain in AC‐ and NaOH‐treated particles (Figure 2) was reduced in patches of secondary wall, between primary and secondary wall, and from cell corners. As xylose is the major sugar detected by sugar analysis (Figure 3), we infer that secondary wall xylans might have contributed to materials extracted by these treatments. However, our results also indicate a role for xylan in cell–cell adhesion. We observed that middle lamellae were removed by sequential treatments of poplar particles using acidic chlorite and dilute alkali (Figure 2) and that xylanase followed by acidic chlorite increased release of single cells (Figure 3). When *A. thaliana* stem material was treated with maleic acid at 160 °C, high‐S‐lignin variants, but not WT or high‐G‐lignin variants, had disrupted middle lamellae (Ciesielski *et al.*, 2014). However, pectins can be extracted by heating alone (Liew *et al.*, 2016; Seixas *et al.*, 2014). We infer that xylans and/or pectins might be cross‐linked to G‐lignin subunits in the CML. Detection of localized xylan subpopulations to wall domains, the middle lamella and the interface between primary and secondary walls is needed to resolve this question.

De‐lignification treatments followed by xylanase were insufficient to effect complete cell separation. In maize coleoptile cell walls, acidic chlorite renders glucuronoarabinoxylan more easily extracted in subsequent treatment with dilute alkali (Carpita, 1983). In poplar wood particles, methylation analysis of the materials extracted by dilute alkali following acidic chlorite treatment showed linkages that are characteristic of both RG‐I and xylans, indicating an association of both RG‐I and xylan with lignin, and perhaps with each other. A complex of glucuronoxylan and RG‐I has been isolated from tomato cell walls (Broxterman and Schols, 2018) and arabinoxylan and RG‐1/HG are covalently linked in the Arabinoxylan Pectin Arabinogalactan Protein1 in Arabidopsis cell walls (Tan *et al.*, 2013). Antibody probes specific to the RG‐I backbone label the middle lamellae of potato tuber cell walls (Buffetto *et al.*, 2015) and in tobacco (*Nicotiana benthianum*) seed endosperm cells, but only after enzymatic digestion of an abundant heteromannan polymer (Lee *et al.*, 2013). Cell–cell adhesion within sieved poplar particles was disrupted entirely by treating de‐lignified particles with xylanase and an RG‐lyase that cleaved the backbone of the RG‐I polymer. In contrast, hydrolysis or lysis of HG did not affect the extent of cell–cell separation. If HG and RG‐I were covalently linked together, then lysis of either component would be expected to disrupt cell–cell adhesion in mature wood. Xylan and RG‐I might mediate cell–cell adhesion before the initiation of lignification in the CML.

### Expression of RG‐lyase facilitates cell–cell separation

Enzymatic or chemical removal of xylan (Cheng *et al.*, 2012) or pectin (Pakarinen *et al.*, 2012) enhances the accessibility of glucanases to cellulose, measured as glucose yield. However, genetic modification of enzymes involved in biosynthesis of the xylan backbone results in decreased stem strength (Brown *et al.*, 2007; Brown *et al.*, 2005; Persson *et al.*, 2007). Over‐expression of *GALACTURONOSYL TRANSFERASE 12* (*GAUT12*) increased recalcitrance to enzymatic digestion but also resulted in decreased growth in *Populus* (Biswal *et al.*, 2018a). Although presumed to be involved in HG backbone synthesis, knockdown of *GAUT12* results in reduced xylan content and in increased growth (Biswal *et al.*, 2015). As observed in several *A. thaliana* cell‐wall mutants with low cell‐wall Ara, Fuc or Rha contents, pleiotropic changes in HG and xylan occur (Mertz *et al.*, 2012). We reasoned that expression of a gene encoding an RG‐lyase might facilitate cell separation without compromising plant growth and development, as RG‐I content is low in wood. Potato tubers expressing a fungal RG‐lyase formed normally, albeit with enlarged cortical cells (Oomen *et al.*, 2002). Arabinan and galactan epitopes, as an indicator of RG‐I localization, accumulated in the middle lamellae of transgenic tubers, in contrast to their primary wall localization in WT tubers (Oomen *et al.*, 2002). In potatoes, adhesion between parenchyma cells is mediated by calcium cross‐linked HG, in common with many fruits and vegetables (Parker *et al.*, 2001). Although differences in stem length or diameter, or leaf number were observed in independent transgenic poplar lines, these were not correlated with the level of transgene expression.

The proportion of single cells released from particles derived from poplar expressing the *AtRGIL6* transgene was correlated with RG‐lyase activity and transcript abundance. In treatments with the Cellic^TM^ Ctec2 cocktail of glucanases and xylanases, *AtRGIL6*‐expressing lines yielded almost twice as much sugar as WT, and treatments to partially separate cells amplified this effect. We infer that expression of RG‐lyase activity is sufficient to increase enzyme accessibility but that surface area can be dramatically increased when partial cell separation is enabled by chemical treatments. We also showed increased fragmentation of sieved particles of high‐expressing lines upon mechanical grinding at a constant speed and for a constant time (Figure S11). Thus, modulation of RG‐I content increases wall porosity to hydrolases at the molecular level, adhesion at the cellular level and the biomechanical properties of particles at the tissue level.

### Implications for the use of poplar as a bioenergy crop

Our results are consistent with previous findings that pectin, although present in low abundance in woody tissues, contributes to biomass recalcitrance, as down‐regulation of pectin synthesis enhanced sugar yield in saccharification assays (Biswal *et al.*, 2018b; Biswal *et al.*, 2015), and over‐expression of *GAUT12* to increase HG content decreased saccharification yield (Biswal *et al.*, 2018a). Deletion of a gene cluster encoding a suite of pectolytic enzymes of *Caldicellulosiruptor bescii* reduced sugar yields in saccharification assays (Chung *et al.*, 2014), whereas controlled expression of recombinant pectinases enhanced digestibility of *A. thaliana* (Tomassetti *et al.*, 2015). Here, we show a novel function for the pectic polysaccharide RG‐I, but not HG, as a determinant of cell–cell adhesion in woody tissue. By determining the molecular basis for cell–cell adhesion in poplar wood, we have identified additional targets for genetic modifications that could substantially reduce energy inputs for biomass processing at the point of use.

## Experimental procedures

### Transgenic plant production and processing

High‐S and ‐G variants were produced in hybrid poplar clone INRA 717‐1B4 (female, *Populus tremula* × *P. alba*) using transgenesis as described previously (Yang *et al.*, 2019). For high‐S lignin variants, cDNA of an *A. thaliana F5H1* gene (*FAH1*, At4g36220) was over‐expressed under control of a promoter from the *A. thaliana C4H* gene (*AtC4H*) (Bell‐Lelong *et al.*, 1997). For high‐G‐lignin variants, RNAi constructs were designed from conserved consensus sequences of the poplar *F5H2* (*P. trichocarpa* ‘Nisqually’ 1/383‐2499) (Yang *et al.*, 2019).

A cDNA of *AtRGIL6* (At2g22620) was cloned using gene‐specific primers RGIL6‐5' (5’‐TGGTGGAGATGAAAGTTGGAG‐3’) and RGIL6‐3' (5’‐TTCTCAAGTTCTAAAAGGACTTTCAAG‐3’) to PCR amplify the full‐length coding sequence of *AtRGIL6* from a cDNA library constructed from total RNA isolated from two‐week‐old *A. thaliana* roots. *AtRGIL6* was sub‐cloned into plant expression binary vector pBl121.

After *Agrobacterium tumefaciens*‐mediated transformation of leaf discs derived from *in vitro*‐cultured plantlets with a strain harbouring one of these binary vectors, plants were regenerated, under selection and rooted shoots were acclimated and grown as described previously (Meilan and Ma, 2006). Transgenic lines expressing *AtRGIL6* were grown in the greenhouse and shoots were harvested for analysis after 2–3 months. The lignin genetic variants of poplar were field‐planted. For processing, stems were oven‐dried at 45 °C for 3 to 7 days, the bark manually peeled with a spoke shave, and the shaved stems subsequently knife‐milled to pass through a ¼" screen by Hazen Research (Golden, CO). Knife‐milled poplar wood was milled further to pass through a 20‐mesh (1 mm) screen of a Wiley Mill (Thomas Wiley, Swedesboro, NJ). Biomass particles were sieved through a nylon mesh (300 µm) to separate particles larger than 300 µm for sequential chemical extraction.

### Cellulose and Klason lignin determinations

Fifteen‐mg samples were suspended in 3 mL of acetic acid/water/nitric acid (8/2/1, v/v/v) in 5‐mL conical glass centrifuge tubes with Teflon^®^‐lined screw caps for hydrolysis of non‐crystalline material at 100 °C for 1 h (Updegraff, 1969). Cellulose was determined as glucose equivalents by a phenol–sulphuric assay (Dubois *et al.*, 1956), using cellulose standards (Sigmacell, Sigma). Klason lignin was determined as described by Kirk and Obst (1988).

### Cell‐separation assays and treatments

Milled‐wood particles (1 g) were suspended in 40 mL of 140 mm acetic acid containing 88 mm sodium chlorite and incubated at 70 °C for 3 h. For the 24‐h chlorite treatments, the suspensions were incubated at 70 °C for 8 h, pelleted by centrifugation, and fresh acidic chlorite was added at each 8‐h timepoint. All supernatants were combined for sugar and linkage determinations. For alkali treatments, 1 g of milled particles were suspended in 40 mL of 0.1 M NaOH, supplemented with 3 mg/mL NaBH_4_, and incubated at ambient temperature for 24 h. For sequential treatments, samples were washed extensively and then treated subsequently with NaOH or acidic chlorite. Wood particles smaller than 300 µm were used for cell–cell separation assays using enzymes, before chemical extraction, to facilitate enzyme diffusion. Wood particles were treated for 24 h with indicated enzymes: endo‐(1→4)‐β‐D‐xylanase M3 from *T. longibrachiatum* (xylanase; pH 6), endo‐(1→5)‐α‐L‐arabinanase (arabinanase; pH 4) from *A. niger*, endo‐(1→4)‐α‐D‐polygalacturonase (PGase; pH 5.5) from *A. aculeatus*, or PL 10 endo‐(1→4)‐α‐D‐polygalacturonan pectate lyase (pectate lyase; pH 10) (http://www.megazyme.com), or PL 11 endo‐(1→4)‐α‐D‐rhamnogalacturonan‐I lyase (RG‐lyase, pH 9) from *C. thermocellum*, according to the manufacturer’s instructions (Nzytech, http://www.nzytech.com). In brief, 5 mg of wood particles were resuspended in 200 µL 50 mm sodium acetate (pH 4, 6, and 5.5); CAPs (pH 10); or Tris‐HCl (pH 9) buffer with 10 units of the corresponding enzyme. Enzyme treatments were carried out in a water bath at 40 °C for 24 h. Samples were washed with water extensively before further treatment, and particles pelleted by centrifugation. Cells and particles were resuspended in water and incubated at 4 °C in 15‐mL conical tubes to determine settled volume.

### Light and transmission electron microscopy

Samples were stained with 1% Toluidine Blue (Fishersci.com) before being mounted on microslides (Gold‐Seal, Fishersci.com). The number of single cells and clusters of 2–4, 5–10 and >10 cells were counted from micrographs obtained using an Olympus BX43 microscope with an Olympus DP26 camera. Proportions of each cluster were determined from greater than 1,000 cells from 20 micrographs from 3 replicate experiments for each genotype and treatment.

For electron microscopy, untreated and treated samples were infiltrated with Embed‐812 epoxy resin (Electron Microscopy Sciences, Hatfield, PA) for 24 h, spun down into a pellet in a 2‐mL vial and polymerized at 70 °C overnight. Semi‐thin toluidine blue‐stained sections were mounted on glass slides for light microscopy (500 nm thickness), then ultrathin sections (200 nm and 100 nm) were collected on 100‐mesh formvar‐coated copper grids, stained with 4% aqueous uranyl acetate, rinsed in water, and finally stained with lead citrate and rinsed. Images were captured with a four megapixel Gatan UltraScan 1000 camera (Gatan, Pleasanton, CA) on an FEI Tecnai G2 20 Twin 200 kV LaB6 TEM (FEI, Hillsboro, OR).

### Chemical Catalytic De‐lignification (CDL) of poplar wood particles

CDL was carried out as described previously (Luo *et al.*, 2016). Briefly, 5 to 15 wt % Ni/C catalyst and 45 mL of methanol were added to 500 mg of transgenic poplar wood particles in a stainless steel Parr reactor, pressurized to 10‐35 bar hydrogen, and heated to 225 °C for 12 h. The reaction mixture was filtered to separate the liquid phase containing aromatic products from the de‐lignified biomass residue.

### Determination of monosaccharide composition and linkage analysis of cell walls and extracted polymers

Cells and cell clusters were pelleted by centrifugation and ground under liquid nitrogen in a mortar and pestle, followed by washes in ethanol and water, to prepare isolated cell walls. Supernatants from acidic chlorite and NaOH extractions were neutralized, dialysed against nanopure water for 48 h, and then freeze‐dried. The carboxyl groups of uronosyl in cell walls or extracted polymers were activated with 1‐cyclohexyl‐3‐(−2‐morpholinyl‐4‐ethyl) carbodiimide (methyl‐*p*‐toluene sulphonate) powder (Sigma‐Aldrich) and reduced with NaBD_4_ (Sigma‐Aldrich) to their respective 6,6‐didueterio sugars (Kim and Carpita, 1992), as modified by Carpita and McCann (1997). These samples were dialysed against deionized water then freeze‐dried. Uronosyl‐reduced samples (1 to 2 mg) were hydrolysed in 1 mL of 2 m trifluoroacetic acid (TFA) containing 0.5 μmol inositol (internal standard) at 120 °C for 90 min, and the supernatant was then evaporated at 40 °C in a stream of air. The sugars were reduced with NaBH_4,_ and alditol acetates were prepared as described previously (Gibeaut and Carpita, 1991). Derivatives were separated by gas–liquid chromatography on a 0.25‐mm × 30‐m column of SP‐2330 (Supelco, Bellefonte, PA). The proportion of 6,6‐dideuteriogalactosyl was calculated using *m/z* 187/189, 217/219 and 289/291 after correction of ^13^C spillover of undeuterated fragments, as described by Kim and Carpita (1992). Partially methylated alditol acetates were prepared (Gibeaut and Carpita, 1991) and gas–liquid chromatography‐electron‐impact mass spectrometry analysis was used to verify all derivative structures (Carpita and Shea, 1989).

### Determination of degree of pectin methyl esterification

Galacturonic acid content of WT cell walls was determined by a carbazole assay in which sulphamate was added to reduce neutral sugar interference and borate was omitted from the H_2_SO_4_ (Filisetti‐Cozzi and Carpita, 1991). For saponification, samples were suspended in 0.75 mL water and 0.25 mL 1.5 m NaOH and incubated at 30 °C for 30 min. The samples were then chilled to ice temperature, and 0.25 mL 4.5 m H_2_SO_4_ was added. After centrifugation, 1.0 mL of supernatant was assayed for methanol as described by Wood and Siddiqui (1971). Degree of methylation was determined by dividing the nmoles of methanol by nmoles of uronic acid.

### Expression analysis

Total RNA was isolated from stems of two‐month‐old poplar plants using the RNeasy Mini Kit according to the vendor’s manual (Qiagen, http://www.qiagen.com). RNA was quantified and treated with TURBO DNA‐free^TM^ DNase (Ambion RNA by Life Technologies, http://www.invitrogen.com). First‐strand cDNA was synthesized from 1 µg of total RNA with the High‐Capacity cDNA reverse transcription kit followed by reverse transcription, according to the vendor’s manual from Applied Biosystems (ThermoFisher, http://www.thermofisher.com). Quantitative RT‐PCR was performed using the StepOne Plus Real‐Time PCR System from Applied Biosystems (ThermoFisher, http://www.thermofisher.com) and Fast Sybr Green Mix from Applied Biosystems (ThermoFisher, http://www.thermofisher.com). The transgene‐specific primers used were as follows: RGIL6 F‐CAA AAC ATT ACC ATC ACG CCA, and RGIL6 R‐GTA CAC CGA TTT CCC ACA ATG. House‐keeping gene (UBQ2) primers used were: UBQ2‐F: TCC AAT GGA ACG GCC ATT AA and UBQ2‐R: TGT ACT CTT TTG AAG TTG GTG T.

### Seed mucilage extraction

Dry *A. thaliana* Col‐0 seeds were immersed in deionized and distilled water and rocked at room temperature for 2‐3 h. Water‐soluble seed mucilage was separated from seeds by centrifugation at 500 ***g*** for 5 min. The supernatant was filtered, transferred to dialysis tubing (Spectra/Por 2 Dialysis Tubing, 12‐14 kD MWCO, Repligen, Rancho Dominguez, CA), dialysed against deionized water and freeze‐dried.

### Rhamnogalacturonan‐I lyase assay

Cell‐wall proteins were isolated from four‐week‐old poplar seedlings grown in MS medium according to Kim *et al. *(2000). Briefly, poplar seedlings were homogenized in ice‐cold extraction buffer (50 mm sodium citrate, 50 mm NaCl, 30 mm ascorbic acid, 1 mm dithiothreitol and 0.1 mm phenylmethylsulfonyl fluoride, pH 6.5), filtered through cheesecloth and the walls washed extensively with citrate buffer (50 mm sodium citrate and 50 mm NaCl, pH 5.5), followed by 20 mm NaCl, and then deionized water. Cell walls were further purified by sequential homogenizations in acetone, 100 mm NaCl, and deionized water. The walls were stirred overnight in ice‐cold 3.5 m LiCl in 20 mm sodium acetate, pH 5.0, and 20 mm NaCl to release basic cell‐wall proteins. The cell‐wall protein solution was concentrated by polyethylene glycol 8000 (Sigma‐Aldrich), and then dialysed against 20 mm sodium acetate, pH 5.0, and 20 mm NaCl. Protein concentration was determined using Quick Start^TM^ Bradford Protein Assay (http://www.Bio-Rad.com).

Rhamnogalacturonan‐I lyase assay was performed with 10 µg cell wall protein in reaction buffer containing 20 mm sodium acetate, pH 5.0, 20 mm NaCl, 5 mm CaCl_2_ and 200 µg of RG‐I substrate, either *A. thaliana* mucilage, Rhamnogalacturonan from soybean pectic fibre (Megazyme), Rhamnogalacturonan‐I from potato pectic fibre (Megazyme, http://www.megazyme.com), or Pectin from citrus fruit (Sigma P9135). Reactions were incubated at 30 °C and absorbance at 235 nm was measured at 30 min, 1, 3, 6, 12 and 24 h.

### Enzymatic digestion of poplar biomass particles

Enzymatic hydrolysis experiments were performed as described previously (Shiga *et al.*, 2017) with 5 mg untreated and treated (3 h acid chlorite followed by 24 h 0.1 m NaOH) wood particles suspended in 2 mL of 50 mm sodium citrate buffer, pH 5, with 1 µL of Cellic™ Ctec2 (1.8 FPU/g particles) at 50 °C in a thermostatically controlled rotary hybridization oven. Eighty % (v/v) ethanol was added to precipitate undigested material in samples, and pelleted by centrifugation for 5 min at 12 000 g. Total sugar contents in supernatants and pellets were determined using the phenol–sulphuric acid method of Dubois *et al. *(1956). Yields of xylose and glucose were determined by preparing alditol acetates as described previously (Gibeaut and Carpita, 1991).

### Pectin extractions

One hundred mg of milled poplar samples in 30‐mL Corex centrifuge tubes were washed with warm (60 °C) 50% ethanol, twice for 10 min each, spun at 5000 g after each wash and rinsed twice with water. Wall material was extracted twice in 10 mL of 0.5% (0.5 g/100 mL) of ammonium oxalate, pH 7.0, at 90 °C in a water bath for 1 h each. After centrifugation, supernatants were combined. Twenty mL of 0.1 m NaOH containing 3 mg/mL NaBH_4_ was added to the pellet and stirred at ambient temperature for 1 h. After centrifugation as above, an additional 10 mL of fresh 0.1 m NaOH was added to the pellet and stirred for 10 min. The ammonium oxalate‐ and 0.1 m NaOH‐extracted materials in supernatants were filtered through GF/F filters by vacuum. NaOH extracts were neutralized with acetic acid. Both ammonium oxalate‐ and NaOH‐extracted materials were dialysed with Barnstead GenPure water (Thermo Fisher Scientific) and freeze‐dried.

### Estimation of proportions of pectic and hemicellulosic polysaccharides from linkage analysis

Using data obtained from methylation analyses, proportions of the RG‐I backbone and its side chains were calculated using their diagnostic linkages. The RG‐I backbone was calculated as the 2‐ and 2,4‐Rha residues plus an equal amount of 4‐GalA. Arabinan was calculated as the mole % sum of 2‐, 3‐ and 5‐Ara, as well as 2,5‐ and 3,5‐Ara branch‐point residues; (type I) galactan was calculated as 4‐ and 3,4‐Gal residues plus *t*‐Ara*f* equal to the 3,4‐Gal. HG and xylogalacturonan (XyHG) were calculated as the remaining 4‐GalA plus 3,4‐GalA and an amount of *t*‐Xyl equal to the branch‐point residue. Type II AGP was calculated as the mole % sum of 3‐, 6‐ and 3,6‐Gal and an amount of *t*‐Ara equal to the branch‐point residue. (Glucurono)xylan was calculated as the mole % sum of 4‐ and 2,4‐Xyl plus the *t*‐GlcA equal to the branch‐point residue. Xyloglucan was calculated as the mole % 4,6‐Glu residues plus 1/3 that amount of 4‐Glu, with *t*‐ and 2‐Xyl equal to the 4,6‐Glu, and *t*‐ and 2‐Gal equal to the amount of 2‐Xyl, and *t*‐Fuc equal to the amount of 2‐Gal. (Gluco)mannan was calculated as the mole % sum of the 4‐ and 4,6‐Man plus an equal amount of 4‐Glc and an amount of *t*‐Gal equal to the 4,6‐Man.

### Mechanical assay

Wood particles of WT and *AtRGIL6*‐expressing lines were sieved to select particles sized between 300 and 500 µm in length. Fifty mg of screened particles were fragmented in a 2‐mL micro‐centrifuge tube with three stainless steel beads using a Spex Certiprep 2000 Geno/Grinder (spexsampleprep.com) at 1500 oscillations per minute for 30 s. Homogenized wood particles were photographed with a Nikon SMZ1500 stereo microscope (nikoninstruments.com). Resulting particle sizes were analysed with a computer program, SmartGrain (Tanabata *et al.*, 2012) set at ‘rough 3’ selectivity.

### Statistical analysis

One‐way analysis of variance (ANOVA), followed by Tukey‐Kramer post hoc pairwise analysis, was used to compare the cell separation, cell‐wall composition and growth phenotypes in WT and transgenic poplar plants. Student’s *t*‐test was used to test for statistical differences of distribution in particle size in mechanical assay and enzymatic digestion of biomass particles between WT and transgenic plants. All statistical calculations were made using Sigma Stat (Systat Software, Point Richmond, CA).

## Authors’ contributions

H.Y., M.R.B., R.M., N.C.C., and M.C.M. designed research; H.Y., M.R.B., R.A.K., A.F., and N.C.C. performed experiments; H.Y., M.R.B., R.M., N.C.C., and M.C.M. analysed data: and H.Y., M.R.B., R.M., N.C.C., and M.C.M. wrote the article.

## Conflict of interests

The authors declare no competing financial interests.

## Supporting information


**Figure S1** Cellulose and lignin contents in wood particles from WT and lignin genetic variants of poplar, and after acidic chlorite and dilute alkali treatments.
**Figure S2** Bright‐field micrographs of particles treated with acidic chlorite (AC) and dilute alkali alone or in combination.
**Figure S3** Release of single cells or clusters of cells from poplar lignin genetic variants after sequential extraction using acidic chlorite and dilute alkali.
**Figure S4** Monosaccharide analyses of materials from WT and lignin genetic variants of poplar extracted in various concentrations of alkali.
**Figure S5** Cell–cell separation of WT and high‐S lignin (S) poplar wood particles after sequential extraction using endo‐(14)‐β‐d‐xylanase (xylanase) and acidic chlorite (AC).
**Figure S6** Cell–cell separation of WT poplar wood particles after sequential extraction using pectic enzymes, acidic chlorite, and dilute alkali alone, or in combination.
**Figure S7** Percentages of cells and cell clusters and release of uronic acids from WT wood particles after treatment with pectolytic enzymes.
**Figure S8** Visible phenotypes of WT and six independent *AtRGIL6*‐expressing poplar lines.
**Figure S9** RG‐lyase activities of cell‐wall proteins isolated from WT and *AtRGIL6*‐expressing plants.
**Figure S10** Relative proportions of polysaccharides extracted from poplar wood of WT and three independent* AtRGIL6*‐expressing lines.
**Figure S11** Expression of *AtRGIL6* in WT poplar facilitates particle fragmentation.
**Table S1** Lignin composition of WT and transgenic poplar milled‐wood particles as determined using Derivatization Followed by Reductive Cleavage (DFRC).
**Table S2** Mass balance of the sequential chemical extractions in cell–cell separation assays of WT and lignin genetic variants of poplar wood.
**Table S3** Linkage analyses of materials extracted from WT and lignin genetic variants of poplar.
**Table S4** Linkage analyses of materials extracted from WT and transgenic poplar wood.
