# Supplementary material for: Rhamnogalacturonan‐I is a determinant of cell–cell adhesion in poplar wood
Source: Plant Biotechnol J. 2019 Oct 23;18(4):1027–40. doi: 10.1111/pbi.13271 (PMC7061878; doi:10.1111/pbi.13271)
Supplement: Supplementary file 1 — Figure S1 Cellulose and lignin contents in wood particles from WT and lignin genetic variants of poplar, and after acidic chlorite and dilute alkali treatments. Figure S2 Bright‐field micrographs of particles treated with acidic chlorite (AC) and dilute alkali alone or in combination. Figure S3 Release of single cells or clusters of cells from poplar lignin genetic variants after sequential extraction using acidic chlorite and dilute alkali. Figure S4 Monosaccharide analyses of materials from WT and lignin genetic variants of poplar extracted in various concentrations of alkali. Figure S5 Cell–cell separation of WT and high‐S lignin (S) poplar wood particles after sequential extraction using endo‐(14)‐β‐d‐xylanase (xylanase) and acidic chlorite (AC). Figure S6 Cell–cell separation of WT poplar wood particles after sequential extraction using pectic enzymes, acidic chlorite, and dilute alkali alone, or in combination. Figure S7 Percentages of cells and cell clusters and release of uronic acids from WT wood particles after treatment with pectolytic enzymes. Figure S8 Visible phenotypes of WT and six independent AtRGIL6‐expressing poplar lines. Figure S9 RG‐lyase activities of cell‐wall proteins isolated from WT and AtRGIL6‐expressing plants. Figure S10 Relative proportions of polysaccharides extracted from poplar wood of WT and three independent AtRGIL6‐expressing lines. Figure S11 Expression of AtRGIL6 in WT poplar facilitates particle fragmentation. Table S1 Lignin composition of WT and transgenic poplar milled‐wood particles as determined using Derivatization Followed by Reductive Cleavage (DFRC). Table S2 Mass balance of the sequential chemical extractions in cell–cell separation assays of WT and lignin genetic variants of poplar wood. Table S3 Linkage analyses of materials extracted from WT and lignin genetic variants of poplar. Table S4 Linkage analyses of materials extracted from WT and transgenic poplar wood. [file PBI-18-1027-s001.pdf]

Table S1 Lignin composition of WT and transgenic poplar milled-wood particles as determined using Derivatization Followed by Reductive Cleavage (DFRC).

	Lignin Composition		
Genotype	H	G*	S
		mole%	
WT (INRA 717-1B4)	0.7±0.0	32.7±0.0	66.6±0.0
High-S (AtC4H:*F5H*)	0.6±0.1	6.3±0.4	93.1±0.3
High-G (AtC4H:*F5H2* RNAi)	0.3±0.5	47.2±0.6	52.4±1.0

*DFRC analysis cannot distinguish between G and 5-OH-G units.

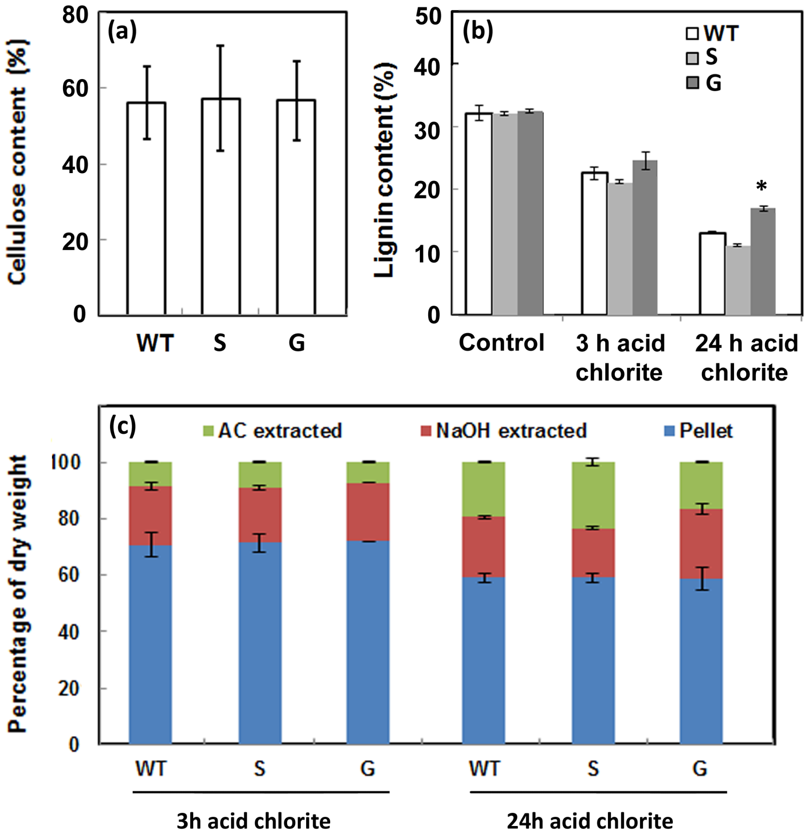

Figure S1 Cellulose and lignin contents in wood particles from WT and lignin genetic variants of poplar, and after acidic chlorite and dilute alkali treatments. (a) Cellulose content as determined by acetic/nitric acid digestion of cell walls. (b) Klason lignin content. (c) Dry weights of materials extracted by treatment with acidic chlorite for 3 h or 24 h, followed by treatment with 0.1 M NaOH for 24 h, and the residual pellet, comprising single cells and cell clusters. WT, wild-type hybrid poplar clone INRA 717-1B4; S, high syringyl-lignin genotype; and G, high guaiacyl-lignin genotype. Values are the means ± SD (n = 3 technical replicates); asterisks indicate significant differences based on Tukey-Kramer Post Hoc test after one-way ANOVA, $p \leq 0.05$ relative to WT.

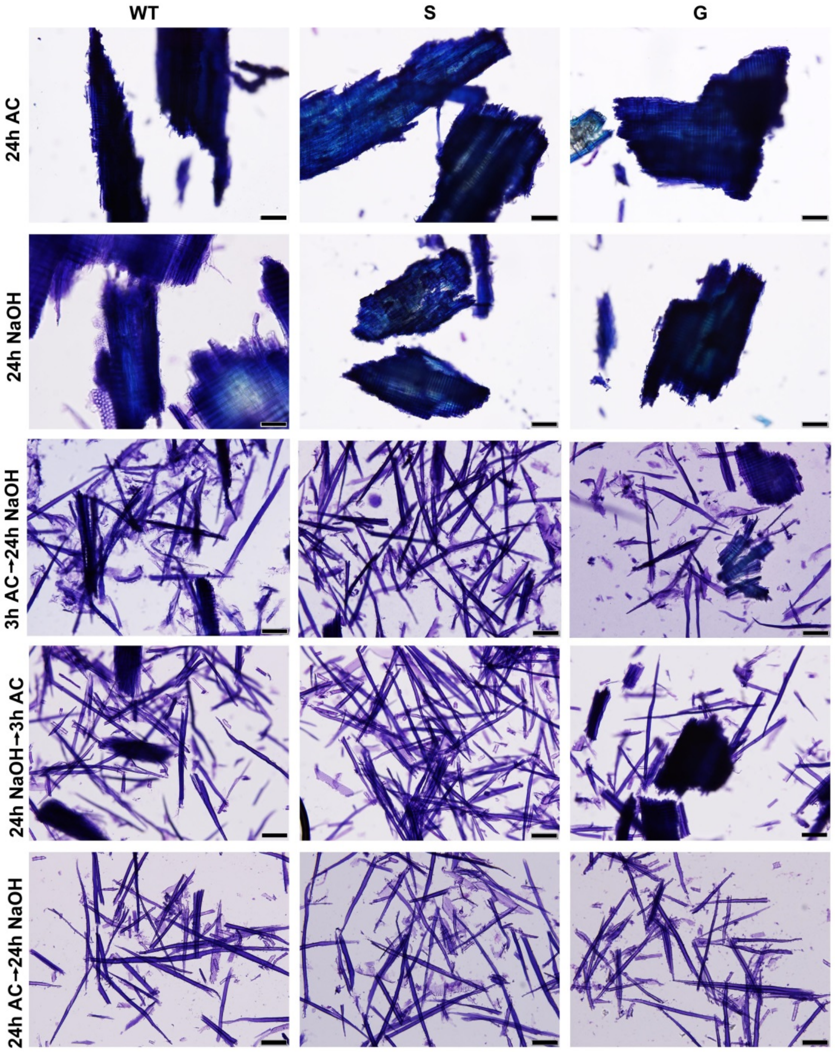

Figure S2 Bright-field micrographs of particles treated with acidic chlorite (AC) and dilute alkali alone or in combination. Treatment with acidic chlorite for 24 h (24 h AC), or 0.1 M NaOH for 24 h (24 h NaOH), alone results in little or no cell separation. Regardless of order, samples treated with acidic chlorite and 0.1 M NaOH show varying degrees of cell-cell separation among wild-type and lignin variants, with high-S lignin (p35S:*F5H*) lines displaying the greatest extent of cell separation. All genotypes show almost complete cell separation after 24 h acidic chlorite treatment followed by 24 h 0.1 M NaOH treatment. WT, wild-type 717-1B4; S, high S-lignin; and G, high G-lignin variant lines. Bar, 100 μm.

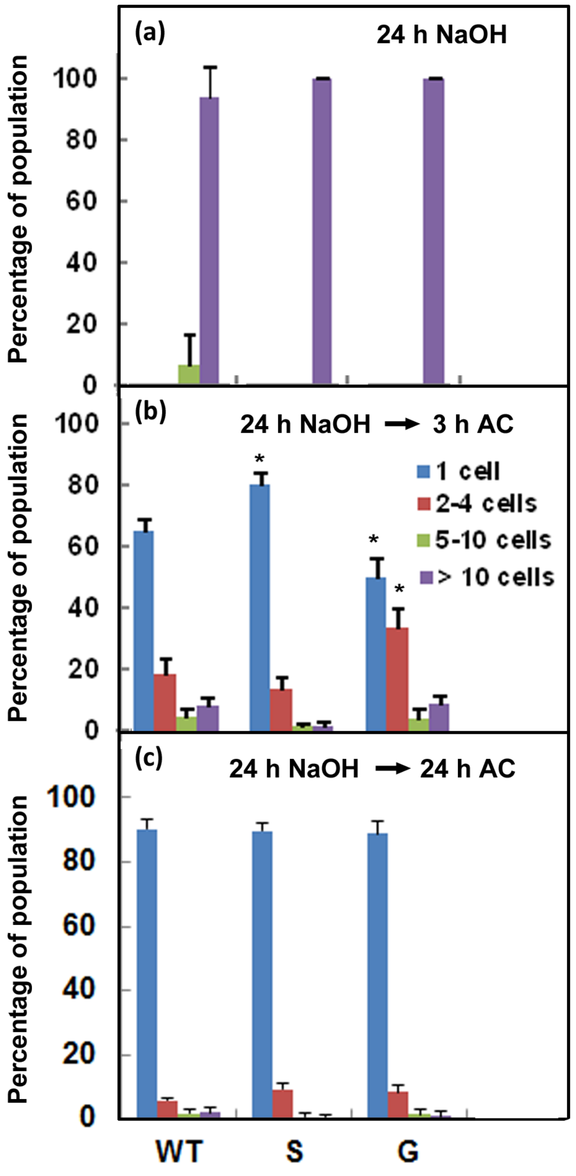

Figure S3 Release of single cells or clusters of cells from poplar lignin genetic variants after sequential extraction using acidic chlorite and dilute alkali. (a) Treatment with 0.1 M NaOH for 24 h (24 h NaOH). (b) Treatment with 0.1 M NaOH for 24 h followed by acidic chlorite for 3 h (24 h NaOH → 3h AC). (c) Treatment with 0.1 M NaOH for 24 h followed by AC for 24 h (24 h NaOH → 24 h AC). WT, wild-type 717-1B4; S, high-S; G, and high-G lignin variant lines. Percentages of single cells (blue) or cell clusters of 2-4 cells (red), 5-10 cells (green), and >10 cells (purple) were determined from >1,000 cells counted per genotype/treatment. Values are the means ± SD (n = 3 technical replicates); asterisks indicate significant differences based on Tukey-Kramer Post Hoc test after one-way ANOVA, $p \leq 0.05$ relative to WT.

Table S2 Mass balance of the sequential chemical extractions in cell-cell separation assays of WT and lignin genetic variants of poplar wood.

Fractions		WT (% DW)	S (% DW)	G (% DW)
**3h acidic chlorite-extracted material**	non-cellulosic sugars	2.2 ± 0.6	2.4 ± 0.1	2.0 ± 0.2
	Lignin	10.0 ± 0.3	11.3 ± 0.2	8.1 ± 1.1
	Protein	0.2 ± 0.0	0.2 ± 0.0	0.2 ± 0.0
**24 h 0.1 M NaOH-extracted material**	non-cellulosic sugars	4.3 ± 0.4	4.0 ± 0.2	4.4 ± 0.1
**Residue**	Lignin	23.4 ± 1.0	21.9 ± 0.5	25.5 ± 1.4
	Cellulose	56.1 ± 1.3	57.2 ± 1.9	56.7 ± 1.4
**Total**		96.1 ± 3.7	97.0 ± 2.9	96.9 ± 4.2

Materials extracted by sequential treatments of acidic chlorite and dilute alkali were dialyzed and freeze-dried. The relative proportions of lignin, protein, cellulose, and non-cellulosic sugars for these materials were determined and calculated as percentages of dry weight (DW) of starting material. WT, wild-type hybrid poplar clone INRA 717-1B4; S, high syringyl-lignin genotype; G, high guaiacyl-lignin genotype. The values are the means ± SD, n = 3 technical replicates.

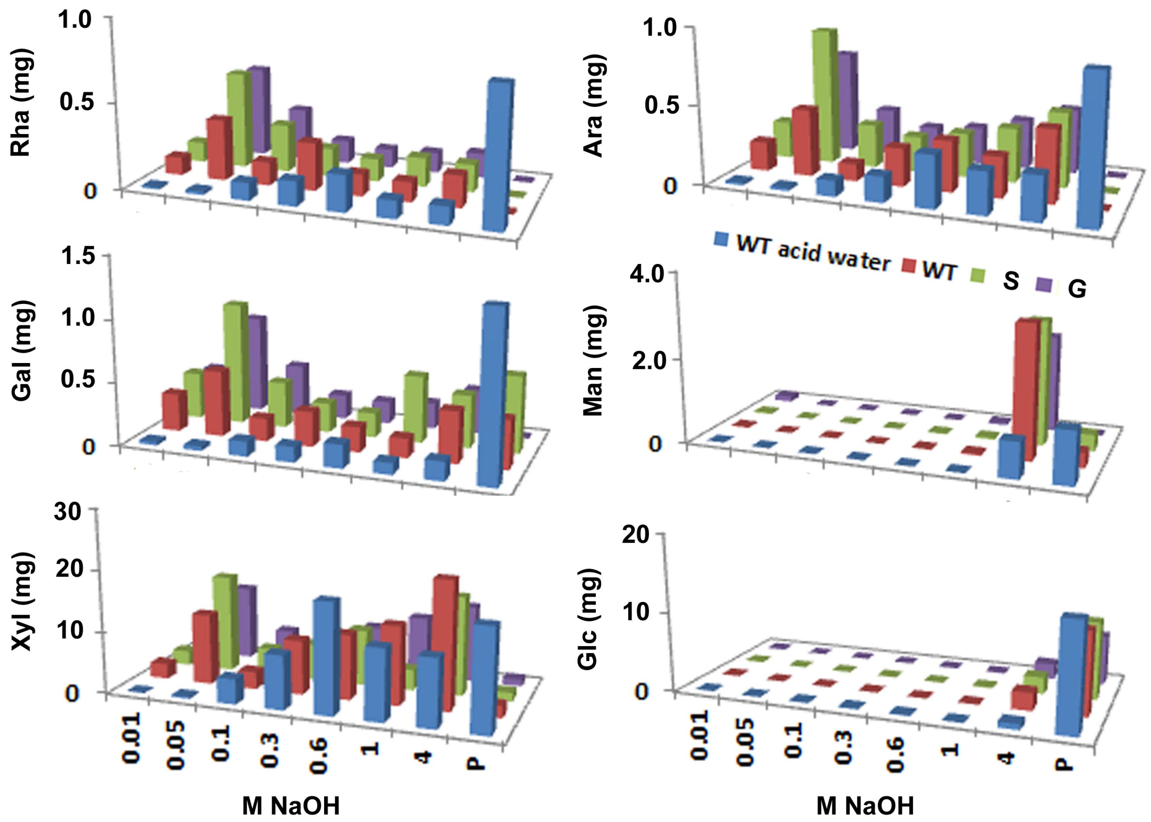

Figure S4 Monosaccharide analyses of materials from WT and lignin genetic variants of poplar extracted in various concentrations of alkali. Values are derived from sugar analyses of alkali-extracted materials from wood particles of acidified-water-treated WT (blue) compared to WT (red), high-S (green), and high-G (purple) lines pre-treated with acidic chlorite for 24 h. Concentrations of alkali are indicated on the x axis, and P indicating the residual pellet after 4M NaOH. WT, wild-type hybrid poplar clone INRA 717-1B4; S, high syringyl-lignin genotype; G, high guaiacyl-lignin genotype.

Table S3 Linkage analyses of materials extracted from WT and lignin genetic variants of poplar. Milled wood particles of wild-type (WT), high-S Lignin (S), and high-G Lignin (G) genotypes were extracted with acidic chlorite (1st step AC) followed by 0.1 M NaOH (2nd step NaOH).[1]

	1st step AC			2nd step NaOH		
Lines	WT	S	G	WT	S	G
			mole %			
t-Rha	0.8	0.9	0.7	0.7	0.6	0.6
2-Rha	2.8	3.3	2.2	1.0	1.0	0.9
2,4-Rha	2.7	2.3	2.1	1.2	1.1	1.1
t-Fuc	4.6	4.5	4.5	1.0	1.1	0.8
t-Ara*f*	3.3	4.1	3.1	1.0	0.2	0.8
t-Ara*p*	1.6	1.7	1.9	0.9	0.2	0.2
2-Ara*f*	0.4	0.4	0.4	0.1	tr	0.1
3-Ara*f*	0.8	1.2	0.8	0.1	3.1	tr
5-Ara*f*	3.0	4.1	2.5	0.6	0.2	0.4
3,5-Ara*f*	1.8	2.0	2.0	1.8	0.4	1.6
t-Xyl*p*	15.1	11.8	20.4	10.5	9.2	8.0
2-Xyl	4.7	5.3	5.4	6.2	6.6	7.1
4-Xyl	12.9	8.9	8.6	46.6	50.8	51.9
2,4-Xyl	18.5	18.7	16.7	14.8	12.3	13.5
3,4-Xyl	0.4	0.3	0.4	1.0	0.8	1.0
4-Man	4.1	4.3	5.6	0.7	0.5	0.7
4,6-Man	0.2	0.1	0.4	n.d.	n.d.	n.d.
t-Gal	3.1	3.2	2.2	0.6	0.6	0.7
2-Gal	0.7	0.7	0.9	0.6	0.5	0.6
4-Gal	0.9	1.2	0.8	0.3	0.3	0.4
6-Gal	1.2	1.2	1.0	0.5	0.5	0.4
2,4-Gal	0.4	0.2	0.3	0.1	0.1	0.2
3,4-Gal	0.3	0.3	0.3	0.1	0.1	0.1
3,6-Gal	1.8	1.9	1.5	1.2	1.0	1.2
t-GalA	0.8	0.8	0.9	1.1	1.2	0.9
4-GalA	2.6	2.9	2.6	3.3	3.2	3.3
2,4 GalA	0.2	0.2	0.2	0.6	0.6	0.7
3,4 GalA	0.6	0.7	0.6	0.5	0.6	0.7
t-GlcA	5.7	6.6	7.4	2.6	2.7	2.4
t-Glc	0.3	0.3	0.5	0.1	0.1	tr
4-Glc	3.2	2.2	2.9	0.2	0.1	0.1
3,4-Glc	0.2	0.1	0.2	tr	0.1	tr
4,6-Glc	0.2	0.3	0.3	0.1	0.1	0.1

[1]Values are means of two independent samples, with less than 5% variance for all linkage groups; tr, trace amounts under 0.05; n.d,, not detected.

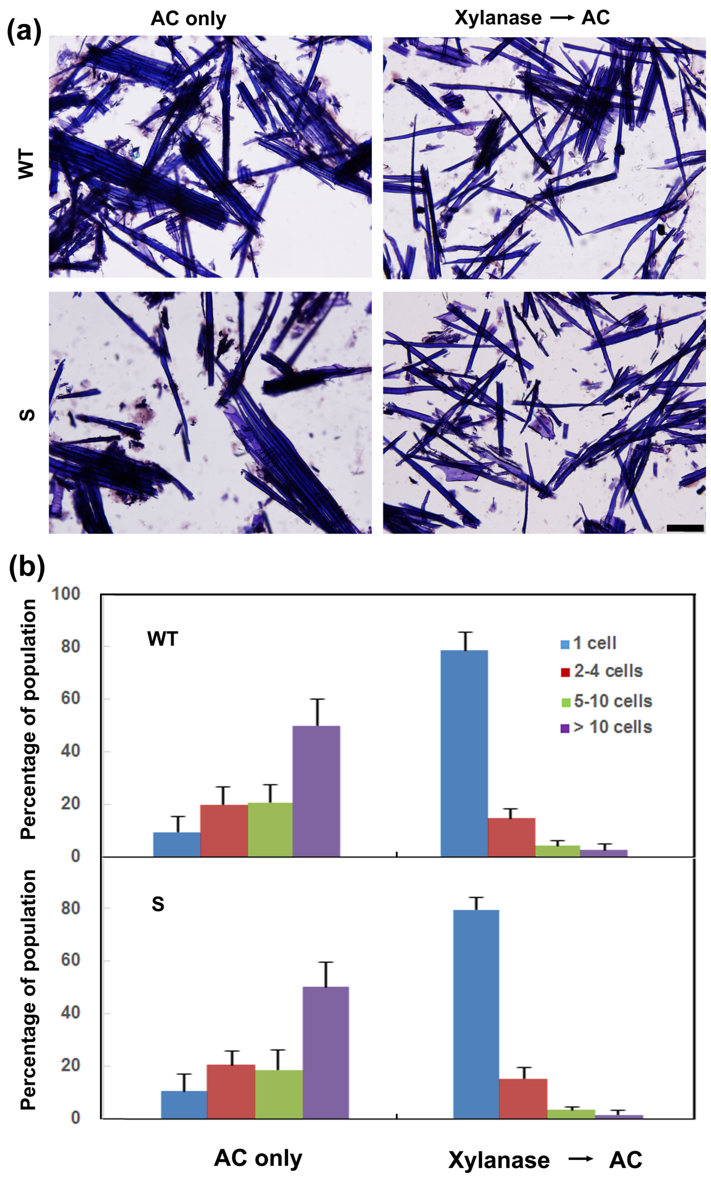

Figure S5 Cell-cell separation of WT and high-S lignin (S) poplar wood particles after sequential extraction using endo-(1→4)-β-d-xylanase (xylanase) and acidic chlorite (AC). (a) Bright-field micrographs of Toluidine Blue-stained wild-type and high-S lignin particles following treatment with acidic chlorite for 24 h (AC only), or treatment with xylanase followed by 24 h acidic chlorite (Xylanase→AC). Bar, 150 µm. (b) Percentages of single cells (blue) or cell clusters of 2-4 cells (red), 5-10 cells (green), and >10 cells (purple) were determined from >1,000 cells counted per genotype/treatment. Values are the means ± SD (n = 3 technical replicates).

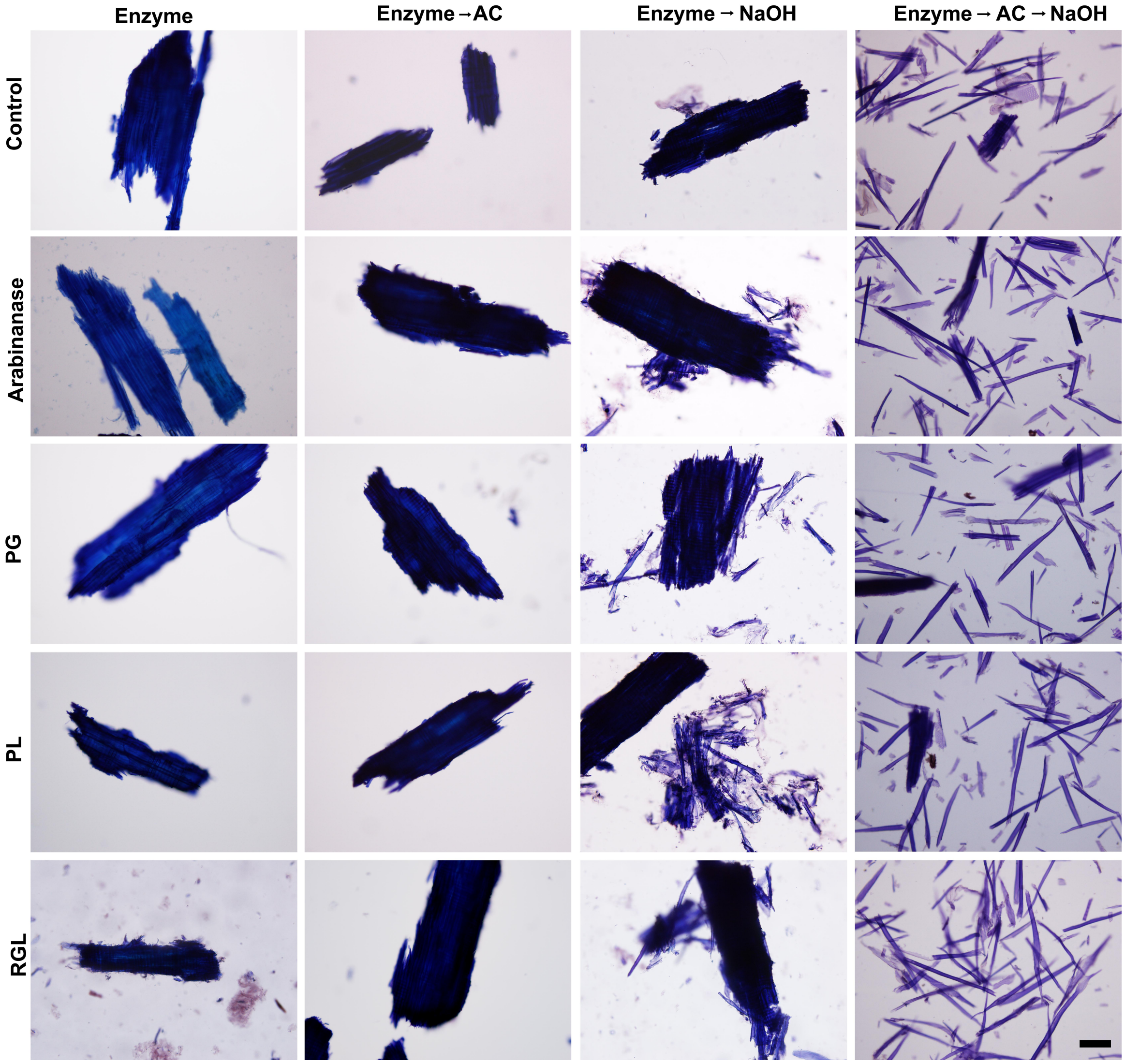

Figure S6 Cell-cell separation of WT poplar wood particles after sequential extraction using pectic enzymes, acidic chlorite, and dilute alkali alone, or in combination. Bright-field micrographs of Toluidine Blue-stained wild-type particles following treatment with buffer (control), arabinanase, polygalacturonase (PG), pectate lyase (PL), or RG-lyase (RGL). Materials were then treated with acidic chlorite for 3 h (Enzyme → AC), 0.1 M NaOH for 24 h (Enzyme → NaOH), or acidic chlorite for 3 h, followed by 0.1 M NaOH for 24 h (Enzyme → AC → NaOH). Bar, 100 μm.

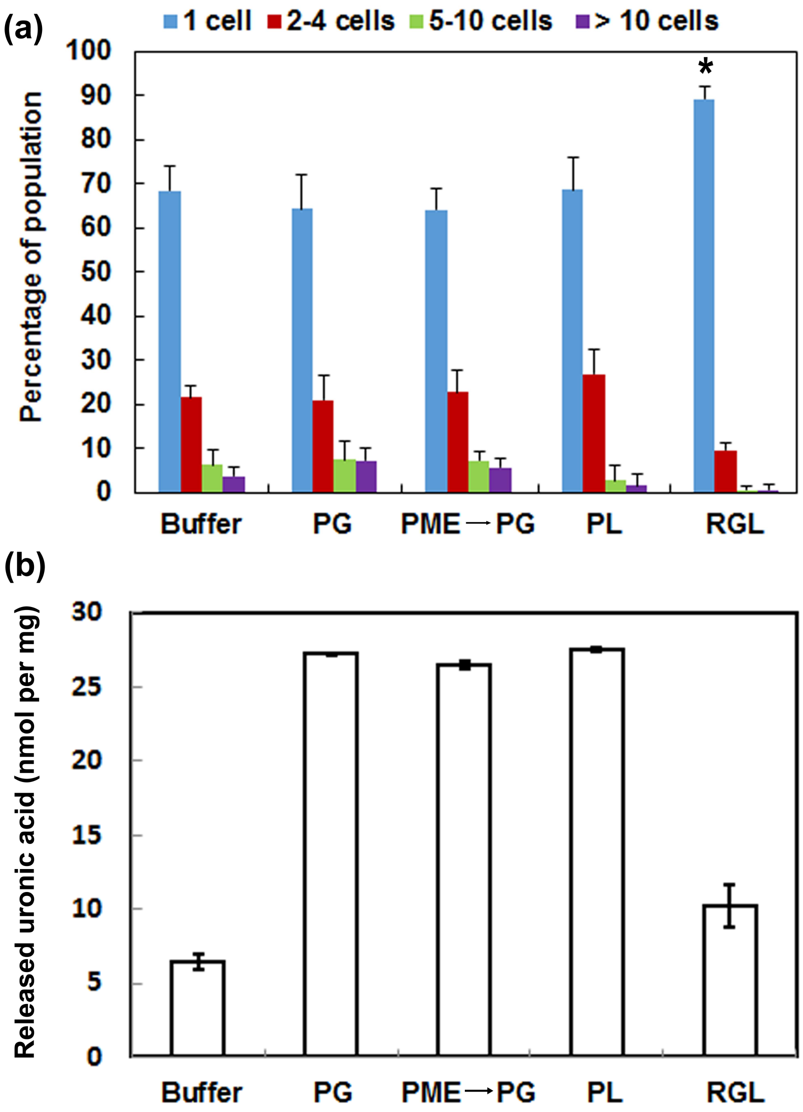

Figure S7 Percentages of cells and cell clusters and release of uronic acids from WT wood particles after treatment with pectolytic enzymes. (a) Polygalacturonase (PG), Pectin Methyl Esterase followed by PG (PME → PG), pectate lyase (PL), or RG-lyase (RGL) treatments were compared to controls without enzyme, and then followed by treatment with acidic chlorite for 3 h and then 0.1 M NaOH for 24 h. Percentages of single cells (blue) or cell clusters of 2-4 cells (red), 5-10 cells (green), and >10 cells (purple) were determined from >1,000 cells counted per genotype/treatment. Values are the means ± SD (*n* = 3 technical replicates); asterisks indicate significant differences based on Tukey-Kramer Post Hoc test after one-way ANOVA, $p \leq 0.05$ relative to WT. (b) Uronic acids released from WT wood particles, nmol per mg of dry weight.

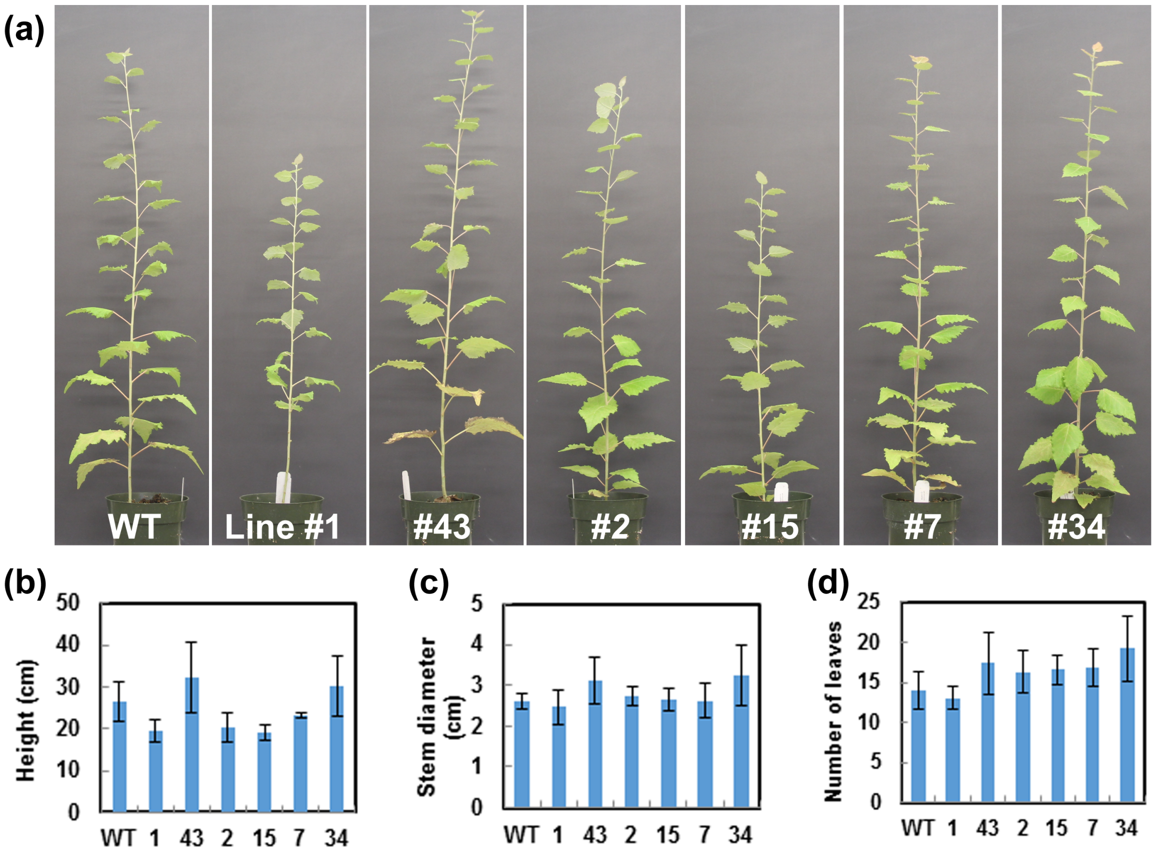

Figure S8 Visible phenotypes of WT and six independent *AtRGIL6*-expressing poplar lines. (a) Two-month-old plants grown in the greenhouse. (b) Height, (c) Stem diameter, and (d) Total number of leaves. Values are the means ± SD, n = 6 biological replicates.

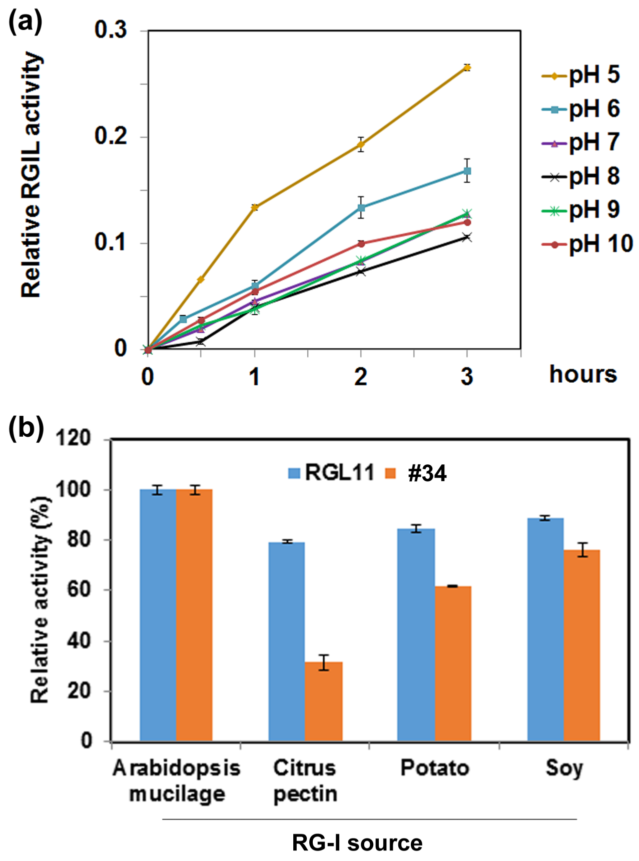

Figure S9 RG-lyase activities of cell-wall proteins isolated from WT and *AtRGIL6*-expressing plants. (a) Relative activity of cell-wall proteins from transgenic line #34 at various pH values. *Arabidopsis thaliana* mucilage was used as RG-I substrate. Values are the means ± SD, *n* = 3 biological replicates. (b) Relative RG-lyase activity of cell-wall proteins from line #34 compared to *Clostridium thermocellum* RGL11, using different sources of pectic fractions containing RG-I. Assay was performed at pH 5.0. Values are the means ± SD, *n* = 3 biological replicates.

Table S4 Linkage analyses of materials extracted from WT and transgenic poplar wood. Milled wood particles from WT poplar and from *AtRGIL6*-expressing lines #15, #7, and #34 were extracted with hot ammonium oxalate (1st step) followed by 0.1 M NaOH (2nd step NaOH). Soluble material recovered after dialysis was carboxyl reduced with $NaBD_4$ to distinguish uronic acids from their respective neutral sugars.[1]

	1st step ammonium oxalate				2nd step NaOH			
Lines	WT	#15	#7	#34	WT	#15	#7	#34
					mole %			
t-Rha	1.3	0.7	0.9	0.6	1.0	1.0	0.5	0.9
2-Rha	2.5	1.8	2.0	1.7	1.7	1.0	1.8	0.9
2,4-Rha	1.2	1.0	1.2	1.4	1.2	0.6	1.0	0.6
t-Fuc	1.0	1.2	0.9	1.2	1.2	1.1	1.1	0.9
t-Ara*f*	7.3	6.1	7.1	5.4	7.9	3.6	6.4	3.3
2-Ara*f*	0.1	0.1	0.1	0.1	0.1	0.1	0.1	tr
3-Ara*f*	1.7	1.1	1.3	1.3	2.4	1.0	2.1	0.9
5-Ara*f*	6.3	5.0	6.0	5.1	5.4	2.0	5.2	2.2
3,5-Ara*f*	2.6	2.6	3.0	2.6	4.1	1.9	3.5	2.2
t-Xyl	1.0	2.1	0.9	2.6	1.9	2.4	1.8	2.1
2-Xyl	0.4	0.9	0.4	1.3	4.6	5.6	5.4	6.8
4-Xyl	0.4	0.8	0.5	1.4	29.3	46.1	34.7	46.6
2,4-Xyl	1.3	3.6	1.2	4.6	7.4	10.9	7.3	10.4
4-Man	1.6	1.5	1.2	1.5	0.2	0.2	0.4	0.2
t-Gal	1.1	1.5	0.4	1.4	2.9	2.2	2.7	2.0
2-Gal	1.7	0.8	3.8	0.8	1.3	1.7	0.8	2.0
4-Gal	2.0	2.1	0.9	2.1	3.9	2.4	4.2	2.4
6-Gal	0.9	0.9	0.5	0.9	0.8	0.8	0.7	0.5
2,4-Gal	1.9	1.6	0.9	1.7	0.7	0.7	0.8	0.4
3,4-Gal	0.3	0.1	0.1	n.d.	0.4	n.d.	n.d.	n.d.
3,6-Gal	2.4	2.1	1.3	2.3	1.9	1.3	1.8	1.2
t-GalA	2.2	2.5	2.8	1.9	1.0	0.7	0.7	0.5
4-GalA	41.1	44.4	45.8	42.5	10.6	6.2	9.5	6.3
2,4-GalA	3.2	1.5	3.6	2.2	0.8	0.7	0.4	0.7
3,4-GalA	5.8	4.4	2.9	4.8	4.0	2.8	3.9	2.5
t-GlcA	0.9	1.6	1.2	1.6	0.7	0.7	1.0	1.0
t-Glc	0.9	1.2	1.5	1.1	0.6	0.5	0.7	0.7
4-Glc	2.7	2.5	2.1	2.4	0.9	0.7	1.0	0.7
3,4-Glc	2.7	2.4	3.8	2.0	0.4	0.4	0.3	0.5
4,6-Glc	1.6	1.9	2.2	1.7	0.6	0.6	0.7	0.7

[1]Values are means of two independent samples, with less than 5% variance for all linkage groups; tr, trace amounts under 0.05; n.d,, not detected.

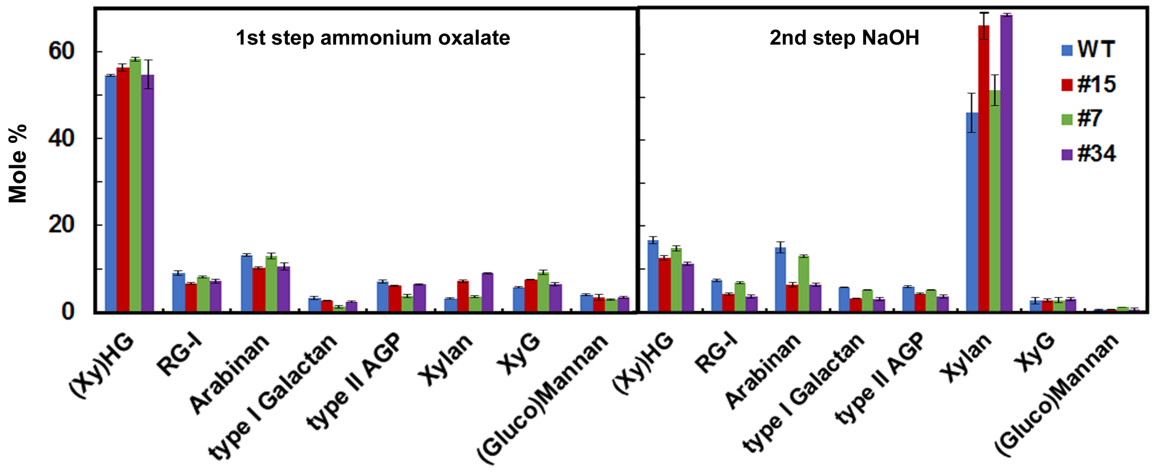

Figure S10 Relative proportions of polysaccharides extracted from poplar wood of WT and three independent *AtRGIL6*-expressing lines. Wood particles from wild-type and *RGIL6*-expressing lines #15, #7, and #34 were extracted twice for 1 h each with hot ammonium oxalate, and the remaining pellet with 0.1 M NaOH. Linkage groups from methylation analysis in Supplemental Table 4 were used to estimate major polysaccharide groups as described in the Materials and Methods. (Xy)HG, xylogalacturonan; AGP, arabinogalactan protein; XyG, xyloglucan.

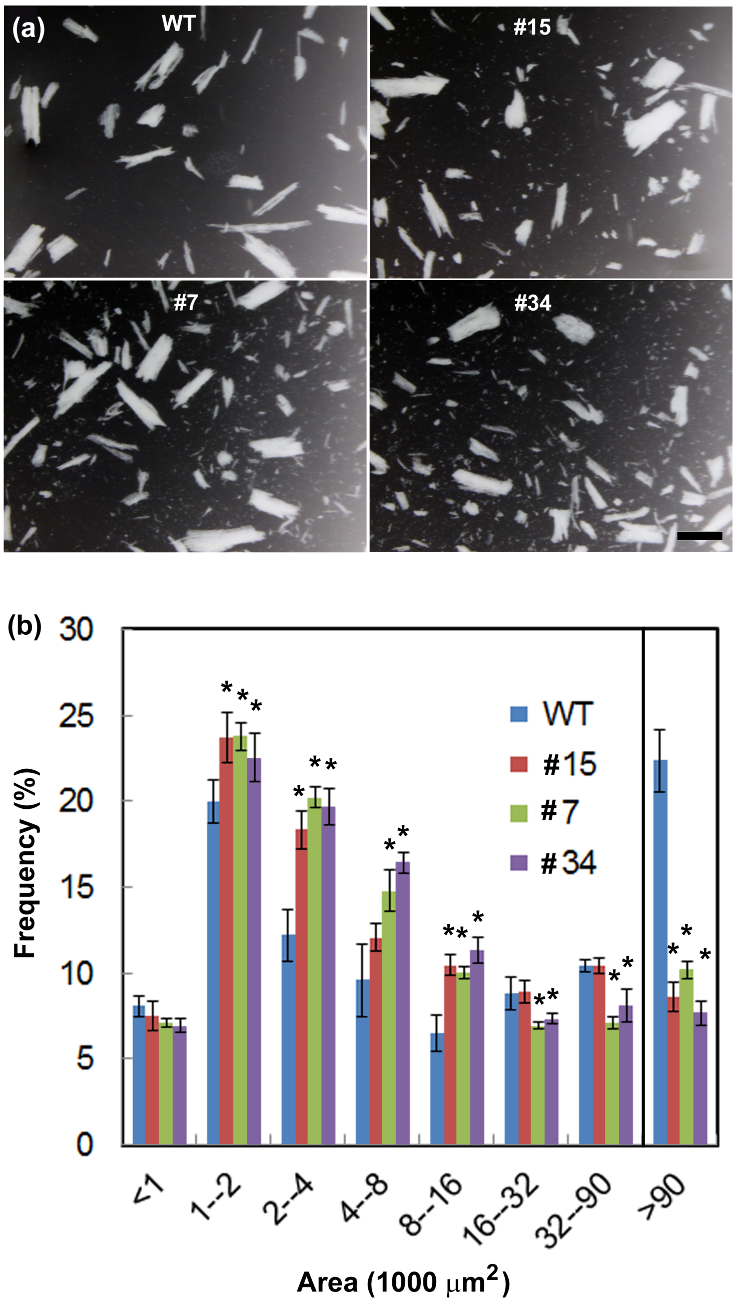

Figure S11 Expression of *AtRGIL6* in WT poplar facilitates particle fragmentation. (a) Micrographs of poplar wood particles from wild-type and three *AtRGIL6*-expressing lines (#15, #7, and #34) after fragmentation using a 'Geno-grinder' cell disruptor operated at 1,500 oscillations per minute for 0.5 min. Bar, 1 mm. (b) Quantification of area of particles after fragmentation. The initial size limit of 90,000 μm^2 is indicated by the black line. Values are the means ± SD (n = 3 biological replicates) of >2,000 particles per genotype. Asterisks indicate significant differences based on Student t-test p <0.05 compared to controls without treatment.
